# Estimates and predictors of health care costs of esophageal adenocarcinoma: a population-based cohort study

**DOI:** 10.1186/s12885-018-4620-2

**Published:** 2018-06-27

**Authors:** Hla-Hla Thein, Nathaniel Jembere, Kednapa Thavorn, Kelvin K. W. Chan, Peter C. Coyte, Claire de Oliveira, Chin Hur, Craig C. Earle

**Affiliations:** 1 Dalla Lana School of Public Health, University of Toronto, Toronto, ON Canada; 2 Institute for Clinical Evaluative Sciences, Toronto, ON Canada; 3 Ottawa Hospital Research Institute, The Ottawa Hospital, Ottawa, ON Canada; 4 School of Epidemiology, Public Health and Preventive Medicine, University of Ottawa, Ottawa, ON Canada; 5 Institute for Clinical Evaluative Sciences (ICES uOttawa), Ottawa, ON Canada; 6 Department of Medicine, University of Toronto, Toronto, ON Canada; 7 Odette Cancer Centre, Sunnybrook Health Sciences Centre, Toronto, ON Canada; 8 Canadian Centre for Applied Research in Cancer Control (ARCC), Toronto, ON Canada; 9 Institute of Health Policy, Management and Evaluation, University of Toronto, Toronto, ON Canada; 10 Centre for Addiction and Mental Health, Toronto, ON Canada; 11 Gastroenterology Division, Massachusetts General Hospital, Boston, MA USA; 12 Harvard Medical School, Boston, MA USA; 13 Canadian Partnership Against Cancer, Toronto, ON Canada

**Keywords:** Costs and cost analysis, Esophageal adenocarcinoma, Health care costs, Stage at diagnosis, Treatment

## Abstract

**Background:**

Esophageal adenocarcinoma (EAC) incidence is increasing rapidly. Esophageal cancer has the second lowest 5-year survival rate of people diagnosed with cancer in Canada. Given the poor survival and the potential for further increases in incidence, phase-specific cost estimates constitute an important input for economic evaluation of prevention, screening, and treatment interventions. The study aims to estimate phase-specific net direct medical costs of care attributable to EAC, costs stratified by cancer stage and treatment, and predictors of total net costs of care for EAC.

**Methods:**

A population-based retrospective cohort study was conducted using Ontario Cancer Registry-linked administrative health data from 2003 to 2011. The mean net costs of EAC care per 30 patient-days (2016 CAD) were estimated from the payer perspective using phase of care approach and generalized estimating equations. Predictors of net cost by phase of care were based on a generalized estimating equations model with a logarithmic link and gamma distribution adjusting for sociodemographic and clinical factors.

**Results:**

The mean net costs of EAC care per 30 patient-days were $1016 (95% CI, $955–$1078) in the initial phase, $669 (95% CI, $594–$743) in the continuing care phase, and $8678 (95% CI, $8217–$9139) in the terminal phase. Overall, stage IV at diagnosis and surgery plus radiotherapy for EAC incurred the highest cost, particularly in the terminal phase. Strong predictors of higher net costs were receipt of chemotherapy plus radiotherapy, surgery plus chemotherapy, radiotherapy alone, surgery alone, and chemotherapy alone in the initial and continuing care phases, stage III-IV disease and patients diagnosed with EAC later in a calendar year (2007–2011) in the initial and terminal phases, comorbidity in the continuing care phase, and older age at diagnosis (70–74 years), and geographic region in the terminal phase.

**Conclusions:**

Costs of care vary by phase of care, stage at diagnosis, and type of treatment for EAC. These cost estimates provide information to guide future resource allocation decisions, and clinical and policy interventions to reduce the burden of EAC.

**Electronic supplementary material:**

The online version of this article (10.1186/s12885-018-4620-2) contains supplementary material, which is available to authorized users.

## Background

Esophageal cancer is the eighth most common cancer worldwide [1]. The incidence of esophageal adenocarcinoma (EAC) has increased rapidly in North America and other Western countries over the past several decades [2–6]. In fact, EAC has become the predominant histological subtype of esophageal cancer (relative to squamous cell carcinoma) in North America and Europe, and the sixth leading cause of cancer-related deaths worldwide [1, 7, 8]. In Canada, the incidence of EAC has risen steadily at 4% per year over the past 30 years (between 1981 and 2009), making it the most common type of esophageal cancer in Ontario [9]. These trends may be attributed to a growing and aging population, and the rise in the prevalence of important risk factors, such as obesity and gastroesophageal reflux disease (GERD) which leads to the development of Barrett’s esophagus [7, 9–11]. Esophageal cancers symptomatically present late and carry poor prognoses, despite advances in multimodality treatment [12, 13]. Esophageal cancer has the second lowest 5-year relative survival rate for people diagnosed with cancer in Canada (i.e., pancreatic cancer 9.5%, esophageal cancer 15.3%, lung cancer 20%, and liver cancer 20.4%) [14]. Therefore, diagnosing esophageal cancers at an early stage before the development of symptoms, is critical for improving prognosis [15].

Recent cancer-related cost estimates placed esophageal cancer patients who survived for more than 1 year post-diagnosis at the top of the cost table at $50,620 (95% CI $47,677–$53,562, 2009 Canadian dollars) [16]. These patients also had the highest cost for hospital admissions of all cancers ($27,506) due to the performance of resource intensive procedures, such as post-surgery esophageal dilation and biopsies to the esophagus or other parts of the gastrointestinal tract (through endoscopies) [16]. Additionally, these patients had frequent post-treatment follow-up visits [17], demonstrated by high costs for physician services ($4757) and home care ($4058) [16]. The costs were higher in the initial and terminal phases, and lower in the pre-diagnosis and continuing phases [18]. However, these studies provide estimates for EAC care that are broad in categorization, and more detailed estimates by specific clinical care elements and characteristic could provide significant data to guide clinical care, policy and future research.

Techniques to reduce EAC incidence, such as endoscopic mucosal resection or radiofrequency ablation of Barrett’s esophagus, will likely be more cost-effective than current surveillance strategies that rely on early detection of cancer [19, 20]. There is, however, limited relevant evidence in the Canadian context; costs estimates of EAC are needed for use in cost-effectiveness analyses of innovative technologies to inform health care professionals, policy makers, and the public in order to aid prevention and the early detection of EAC.

The purpose of this study was to estimate: i) the phase-specific net direct medical costs of care attributable to EAC for all adults aged 18 years and older, from the perspective of the Ontario Ministry of Health and Long-Term Care; ii) total net health care costs by cancer stage and type of treatment for EAC; and iii) predictors of the total net costs of care for individuals diagnosed with EAC.

## Methods

### Study design and setting

We conducted a population-based retrospective cohort study by linking the Ontario Cancer Registry (OCR) with administrative health data and a reference Ontario population to estimate the phase-specific net costs of care for primary EAC from January 1, 2003, through December 31, 2011. Individuals were followed from the day of diagnosis until death or until 12 months after the end of the study period, i.e., December 31, 2012, whichever came first. We approached costing [21–25] based on three care phases: 1) initial phase, the first 12 months after diagnosis of EAC, which would include diagnostic services, primary therapy, and adjuvant therapy to lower the risk of cancer recurrence; 2) continuing care phase, all months between the initial and terminal phases of care, which would include surveillance activities for detecting recurrences, follow-up treatment to prevent cancer recurrence, and treatment of complications following the initial therapy; and 3) the terminal phase, the final 12 months before death, which applies to care received at the end of life, often palliative in nature. For patients who died within 12 months post-diagnosis, the costs were attributed to the terminal phase only. For patients surviving < 24 months after diagnosis, the final 12 months of observation and costs of care were allocated to the terminal phase first while the remaining months were allocated to the initial phase [21, 25]. For patients who did not die during the study period, the first 12 months (and costs) were allocated to the initial phase and all remaining months were allocated to continuing care phase [22]. We estimated phase-specific net costs of care as the difference between the mean costs for EAC cases and for matched controls without cancer [22, 24, 25]. Additionally, we stratified total net costs by stage at diagnosis and treatment for EAC, and identified predictors of total net costs.

### Data sources

We conducted our analyses using population-level administrative health databases with information on all 14 million Ontario residents. Data were provided by the Institute for Clinical Evaluative Sciences, the main data repository for health records in the province of Ontario, Canada. These data have been validated for completeness and accuracy [26–31]. This included cancer registry linked to demographic and geographic information, physician billings for outpatient, inpatient, community-based, and laboratory services, hospital and emergency department discharge abstracts, hospital-based ambulatory care data, and prescription drugs (for those over age 65), home care, continuing care, and long-term care [32, 33].

All cancer incidence in Ontario and subsequent mortality has been captured by the OCR from 1964 onwards. The Registered Persons Database contains demographic and geographic information for all people registered for provincial government-sponsored health insurance coverage. The Ontario Health Insurance Plan (OHIP) claims database contains the records of all physician billings for outpatient, inpatient, community-based, and laboratory services starting from July 1991. Non-physician procedures with an OHIP billing number (for example, midwife, chiropractor, nurse practitioner, or physiotherapist) are also included. Billings are based on the Ontario Health Insurance Plan fee-for-service rates in effect in the year the services were provided. The Canadian Institute for Health Information Discharge Abstract Database (CIHI-DAD) contains demographic, clinical, and administrative information on inpatient hospitalizations from April 1988 onwards; and CIHI-National Ambulatory Care Reporting System (CIHI-NACRS) contains administrative, demographic, clinical, and financial data for hospital-based and community-based ambulatory care (day surgery, emergency department visits, outpatient and community-based clinics) which is available from April 2003 onwards. OHIP, CIHI-DAD, and CIHI-NACRS fee codes were used to identify surgical resection, chemotherapy and radiotherapy, as well as esophageal dilation, drainage, esophageal stenting, laser debulking of tumor, and palliative care for EAC (see Additional file 1: Table S1). We used previously published and validated fee codes for these procedures [34].

Direct medical costs were determined using the perspective of the public payer. The costing methods followed the guidelines of the Canadian Agency for Drugs and Technology in Health [33] and the Health System Performance Research Network [32], and were based on previous cancer costing work done in Ontario [16, 18, 35]. Costs associated with physician services, including outpatient visits, laboratory services, diagnostic tests, emergency physicians, and medical and radiation oncologists, were determined through the OHIP claims database. The cost of inpatient hospitalization was determined from the CIHI-DAD database. Costs associated surgical resection, chemotherapy and radiotherapy for EAC were determined using the CIHI and OHIP databases with application of standard provincial unit costs. Emergency department visit and same-day surgery costs came from the CIHI-NACRS database. Ontario Drug Benefit Program database contains the cost of prescription medication dispensed to individuals 65 years of age and older, resident of a long-term care facility or a home for special care, recipient of services under the Home Care Program, recipient of social assistance (Ontario Works, Ontario Disability Support Program), registered under the Trillium Drug Program, or registered under the Special Drugs Program. The Ontario Home Care Services, Continuing Care Reporting System, and OHIP/Ontario Drug Benefit Program databases were used to identify costs associated with home care, continuing care (chronic care), and long-term care.

### Study variables

Variables considered in the analyses included sociodemographic characteristics: age group at diagnosis (< 50, 50–54, 55–59, 60–64, 65–69, 70–74, 75–79, 80–84, ≥ 85 years); gender (male, female); residence (rural, urban); birth country (outside of Canada, Canada); area-level income quintile (Q1-lowest; Q5-highest); Ontario administrative health region (Erie St. Clair, South West, Waterloo Wellington, Hamilton Niagara Haldimand Brant, Central West, Mississauga Halton, Toronto Central, Central, Central East, South East, Champlain, North Simcoe Muskoka, North East, North West); and clinical characteristics such as comorbidity, measured by the Johns Hopkins Aggregated Diagnosis Groups (number of ADGs: 0, 1–3, 4–7, 8–10, 11+); stage at EAC diagnosis (Stage 0-earliest stage of EAC, also called high-grade dysplasia, where cancer cells are found only in the epithelium, Stage I, Stage II, Stage III, Stage IV); treatment for EAC (categorized exclusively as surgery, chemotherapy or radiotherapy alone, surgery plus chemotherapy, surgery plus radiotherapy, chemotherapy plus radiotherapy, surgery plus chemotherapy plus radiotherapy, and no treatment); year of EAC diagnosis (2003–2011); and date of death. The OCR has used the American Joint Committee on Cancer TNM staging [36] from 2003 onwards.

Individual-level income quintile was not available; therefore, area-level income quintile was used as a surrogate. Area-level income quintile was quantified using median neighbourhood household income, which was determined through linking of postal codes to Canadian census data and categorized into quintiles corresponding to income status of neighbourhoods. The income quintile 1 represents the lowest 20% of neighbourhoods and income quintile 5 represents the most well-off 20% of neighbourhoods.

Ontario has 14 health regions, called Local Health Integration Networks (LHIN) [37] which we used as a factor to explain regional health care service and availability. The Johns Hopkins Adjusted Clinical Groups case-mix system [38–41] was used for comorbidity adjustment [42–44].

### Estimates of the net cost of care for EAC patients: Matching cases and controls

The net cost method matches cases and controls on socio-demographic and clinical factors associated with resource use and calculates the difference in cost for cancer patients and non-cancer control subjects [22, 24, 25]. Cases (cancer patients) were identified as all eligible individuals 18 years of age and older in the OCR with an International Statistical Classification of Disease and Related Health Problems (ICD-9) site codes 150.0–150.9 and ICD-10 codes (C15.3–C15.9), in combination with histology International Classification of Diseases for Oncology, Third Edition (ICD-O-3) codes 8140–8575 corresponding to primary cancer (see Additional file 1: Table S2) [45]. Individuals were excluded if the EAC diagnosis was recorded on or after the date of death or individuals whose EAC was not the primary site.

Potential controls were selected from a 5% random sample of the reference Ontario population Registered Persons Database, including all individuals 18 years of age and older with no cancer diagnosis before or during our analysis period. Control subjects who died before the patient’s EAC diagnosis date were excluded.

Two sets of cases and controls were used to match 1:1 at two index dates (date of diagnosis and 12 months preceding the date of death) to estimate costs for the initial and continuing care phases. For the latter index date, cases who died were matched 1:1 to controls with similar conditional probability of a diagnosis of EAC given the observed individual covariates [46, 47] who died on the same date to estimate costs for the terminal phase. This was derived by fitting a logistic model with EAC status as the dependent variable and the index year (year of EAC diagnosis), age group at index date, gender, urban or rural residence, neighbourhood income quintile, Ontario health region, and comorbidity [18, 35]. For each case, the closest non-EAC control was selected that matched the following criteria: age ± 5 years at the index date; same gender; same index year; comorbidity (ADGs), and a propensity score within a caliper width of 0.2 standard deviation [48].

### Estimation of health care costs

Cost estimates for inpatient hospitalizations, same-day surgery, and emergency department visits were obtained by multiplying the resource intensity weight (measure of resource utilization intensity) by the cost per weighted case (unit cost) [32, 49–51]. Costs for services included in Ontario Health Insurance Plan, Ontario Drug Benefit, and Home Care were obtained by multiplying the number of services by unit cost. Continuing care cost was determined using Continuing Care Reporting System, which contains clinical and demographic information on individuals receiving facility based continuing care. Services include medical long-term care, rehabilitation, geriatric assessment, respite care, palliative care, and nursing home care. Patients are classified into 44 Resource Utilization Groups, and are assigned a Case Mix Index that approximates their per day resource usage. Case Mix Index is reviewed every quarter and can be adjusted multiple times [32]. Continuing care cost per weighted day was derived by dividing the total annual cost by the total annual weighted day. The case cost is the product of weighted days multiplied by the cost per weighted day. The cost of long-term care was obtained through the product of the year-specific length of stay and the Ministry of Health cost per diem. All costs were adjusted to 2016 Canadian dollars using the Consumer Price Index for Health and Personal Care [52]. Costs were undiscounted (i.e., exact costs billed).

### Statistical analysis

Sociodemographic and clinical characteristics and health care costs for the EAC cases and non-EAC control cohorts were summarized by phase of care. We presented categorical variables as frequencies and percentages, and continuous variables as means ± standard deviations. For each phase of care, we estimated mean (95% confidence interval [CI]) net costs of care due to EAC (per 30 patient-days) using generalized estimating equations to account for the matched study design. Estimates were bootstrapped 1000 times to obtain CIs. Total net health care costs and by phase of care were analyzed by stage at EAC diagnosis and type of treatment received.

Generalized estimation equation model with a logarithmic link and gamma distribution, which specifies the conditional mean function directly, was used to examine unadjusted and adjusted relationships between covariates and total net health care costs per 30 patient-days by phase of care among all EAC cases [53–55]. Potential covariates included age at EAC diagnosis, gender, urban or rural residence, birth country, income quintile, Ontario health region, comorbid conditions (ADGs), stage of disease at diagnosis, treatment for EAC, and year of EAC diagnosis. Variables with a significance level of *P* ≤ 0.2 in the univariate analyses were entered into the multivariate generalized estimation regression analysis and were considered independently significant when *P* ≤ 0.05 [56, 57]. Interactions were considered in the context of regression analysis. The adjusted model was constructed according to a stepwise backward selection methodology and only included those variables that remained significant at the two-sided level of *P* ≤ 0.05 [57]. Finally, variables that were non-significant in the univariate test were added to see if they became significant when adjusted for other factors [58]. Statistical analyses were conducted using SAS version 9.4 (SAS Institute Inc., Cary, NC, USA).

#### Sensitivity analysis

A sensitivity analysis was performed where the initial phase was defined as the first 6 months after diagnosis of EAC, the terminal phase was defined as the final 6 months before death, and the continuing care phase was defined as all months between the initial and terminal phases of care.

## Results

### Characteristics of the study population

A flow chart of the study population is shown in Additional file 2: Figure S1. Overall, 3035 EAC cases and 560,997 control subjects were identified during the study period 2003–2011 (see Additional file 3: Table S3). Over the period, the number of new EAC cases increased from 285 in 2003 to 413 in 2011, and the proportion of those with age group at diagnosis of 50–54, 55–59, 60–64, 65–69, and 70–74 years increased from 5.8, 10.5, 8.3, 8.0, and 10.3% to 12.6, 17.1, 13.9, 18.3, and 12.5%, respectively. Stage at EAC diagnosis was available from 2003 in the data; 126 (4.2%) people were diagnosed with stage 0-I, while 420 (13.8%) were stage II, 455 (15.0%) were stage III, 940 (31.0%) were stage IV, and 1094 (36.1%) were unknown stage. In addition, the proportion of patients with known stages increased from 2003 to 2011; stage 0-I from 1.6 to 21.4%; stage II from 3.1 to 12.6%; stage III from 0.9 to 16.0%; and stage IV from 2.5 to 11.5%. Patients receiving treatment with radiotherapy alone after EAC diagnosis increased from 5.2% in 2003 to 19.3% in 2011. In addition, those not receiving treatment increased from 8.9 to 14.0%. In contrast, the proportion of patients receiving surgery plus chemotherapy decreased over time, from 13.6 to 5.1%. In our cohort, 2490 of EAC patients died during the mean 510 days or median 288 days of follow-up and 18,536 of controls died during the mean 2309 days or median 2373 days of follow-up.

Table 1 describes the baseline characteristics of the matched cases and controls by phase of care. Cases that contributed person-time to the initial (259 days) and the terminal phase (242 days) were closely matched to the controls (initial phase: 360 days and terminal phase: 360 days); however, many cases that contributed person-time to the continuing care phase could not be matched with suitable controls (726 versus 1521 days).

**Table 1:** Matched cases (esophageal adenocarcinoma) and controls by phase of care, 2003–2011

Variable	Initial Phase		Continuing Care Phase		Terminal Phase	
	Cases	Controls	Cases	Controls	Cases	Controls
	N (%)	N (%)	N (%)	N (%)	N (%)	N (%)
N	1265	1265	632	632	3011	3011
Mean ± SD time spent (days)	259 ± 130	360 ± 34	726 ± 691	1521 ± 892	242 ± 130	360 ± 36
Age group at index date (years)						
< 50	106 (8.4)	106 (8.4)	53 (8.4)	53 (8.4)	242 (8.0)	242 (8.0)
50–54	138 (10.9)	138 (10.9)	76 (12.0)	76 (12.0)	277 (9.2)	277 (9.2)
55–59	165 (13.0)	165 (13.0)	84 (13.3)	84 (13.3)	331 (11.0)	331 (11.0)
60–64	227 (17.9)	227 (17.9)	116 (18.4)	116 (18.4)	459 (15.2)	459 (15.2)
65–69	203 (16.1)	203 (16.1)	113 (17.9)	113 (17.9)	423 (14.1)	423 (14.1)
70–74	161 (12.7)	161 (12.7)	86 (13.6)	86 (13.6)	413 (13.7)	413 (13.7)
75–79	138 (10.9)	138 (10.9)	66 (10.4)	66 (10.4)	383 (12.7)	383 (12.7)
80–84	79 (6.3)	79 (6.3)	24 (3.8)	24 (3.8)	279 (9.3)	279 (9.3)
≥ 85	48 (3.8)	48 (3.8)	14 (2.2)	14 (2.2)	204 (6.8)	204 (6.8)
Gender						
Female	198 (15.7)	198 (15.7)	86 (13.6)	86 (13.6)	499 (16.6)	499 (16.6)
Male	1067 (84.4)	1067 (84.4)	546 (86.4)	546 (86.4)	2512 (83.4)	2512 (83.4)
Residence						
Urban	1033 (81.7)	1045 (82.6)	518 (82.0)	529 (83.7)	2464 (81.8)	2498 (83.0)
Rural	232 (18.3)	220 (17.4)	114 (18.0)	103 (16.3)	547 (18.2)	513 (17.0)
Income quintile						
Q1 (lowest)	239 (18.9)	250 (19.8)	109 (17.3)	118 (18.7)	598 (19.9)	636 (21.1)
Q2	247 (19.5)	251 (19.8)	111 (17.6)	121 (19.2)	629 (20.9)	616 (20.5)
Q3	242 (19.1)	228 (18.0)	121 (19.2)	108 (17.1)	587 (19.5)	566 (18.8)
Q4	274 (21.7)	278 (22.0)	140 (22.2)	139 (22.0)	623 (20.7)	646 (21.5)
Q5 (highest)	263 (20.8)	258 (20.4)	151 (23.9)	146 (23.1)	574 (19.1)	547 (18.2)
Ontario health region						
Erie St. Clair	65 (5.1)	65 (5.1)	35 (5.5)	37 (5.9)	153 (5.1)	149 (5.0)
South West	88 (7.0)	89 (7.0)	40 (6.3)	42 (6.7)	273 (9.1)	275 (9.1)
Waterloo Wellington	72 (5.7)	73 (5.8)	31 (4.9)	33 (5.2)	174 (5.8)	169 (5.6)
Hamilton Niagara Haldimand Brant	174 (13.8)	178 (14.1)	80 (12.7)	84 (13.3)	445 (14.8)	450 (15.0)
Central West	51 (4.0)	47 (3.7)	31 (4.9)	30 (4.8)	101 (3.4)	89 (3.0)
Mississauga	56 (4.4)	57 (4.5)	26 (4.1)	26 (4.1)	140 (4.7)	145 (4.8)
Toronto Central	78 (6.2)	82 (6.5)	38 (6.0)	40 (6.3)	199 (6.6)	207 (6.9)
Central	103 (8.1)	103 (8.1)	59 (9.3)	59 (9.3)	214 (7.1)	215 (7.1)
Central East	138 (10.9)	134 (10.6)	72 (11.4)	67 (10.6)	342 (11.4)	339 (11.3)
South East	94 (7.4)	89 (7.0)	48 (7.6)	44 (7.0)	210 (7.0)	203 (6.7)
Champlain	168 (13.3)	170 (13.4)	83 (13.1)	79 (12.5)	338 (11.2)	351 (11.7)
North Simcoe Muskoka	64 (5.1)	70 (5.5)	34 (5.4)	40 (6.3)	146 (4.9)	155 (5.2)
North East	79 (6.3)	78 (6.2)	38 (6.0)	35 (5.5)	193 (6.4)	194 (6.4)
North West	35 (2.8)	30 (2.4)	17 (2.7)	16 (2.5)	83 (2.8)	70 (2.3)
ADGs						
0	4 (0.3)	4 (0.3)	3 (0.5)	3 (0.5)	15 (0.5)	15 (0.5)
1–3	49 (3.9)	49 (3.9)	21 (3.3)	21 (3.3)	117 (3.9)	117 (3.9)
4–7	235 (18.6)	235 (18.6)	118 (18.7)	118 (18.7)	549 (18.2)	549 (18.2)
8–10	341 (27.0)	341 (27.0)	155 (24.5)	154 (24.5)	803 (26.7)	803 (26.7)
11+	636 (50.3)	636 (50.3)	335 (53.0)	335 (53.0)	1527 (50.7)	1527 (50.7)
Year of EAC diagnosis						
2003	118 (9.3)	118 (9.3)	59 (9.3)	57 (9.3)	284 (9.4)	284 (9.4)
2004	126 (10.0)	126 (10.0)	74 (11.7)	74 (11.7)	288 (9.6)	288 (9.6)
2005	97 (7.7)	97 (7.7)	63 (10.0)	63 (10.0)	279 (9.3)	279 (9.3)
2006	139 (11.0)	139 (11.0)	71 (11.2)	71 (11.2)	320 (10.6)	320 (10.6)
2007	122 (9.6)	122 (9.6)	64 (10.1)	64 (10.1)	297 (9.9)	297 (9.9)
2008	139 (11.0)	139 (11.0)	81 (12.8)	81 (12.8)	349 (11.6)	349 (11.6)
2009	165 (13.0)	165 (13.0)	90 (14.2)	90 (14.2)	386 (12.8)	386 (12.8)
2010	181 (14.3)	181 (14.3)	85 (13.5)	85 (13.5)	397 (13.2)	397 (13.2)
2011	178 (14.1)	178 (14.1)	45 (7.1)	45 (7.1)	411 (13.7)	411 (13.7)

*SD* standard deviation, *ADGs* Aggregated Diagnosis Groups, *EAC* esophageal adenocarcinoma

### Phase-specific health care costs and net costs of care

The average total health care costs per 30 patient-days among EAC patients was relatively high in the initial phase ($1139; 95% CI, $1079–$1199), declined during the continuing care phase ($923; 95% CI, $852–$995), and increased markedly in the terminal phase ($9004; 95% CI, $8545–$9462) (Table 2).

**Table 2:** Mean health care costs^a^ among EAC cases and non-EAC controls according to service category and phase of care, 2003–2011

Service category	Initial Phase		Continuing Care Phase		Terminal Phase	
	Cases	Controls	Cases	Controls	Cases	Controls
	Mean (95% CI)	Mean (95% CI)	Mean (95% CI)	Mean (95% CI)	Mean (95% CI)	Mean (95% CI)
N	1285	2942	651	2870	3011	3011
Outpatient visits	$170 ($164–$177)	$16 ($15–$17)	$157 ($150–$165)	$40 ($38–$42)	$1263 ($1199–$1327)	$37 ($25–$49)
Emergency department visits	$20 ($19–$22)	$3 ($3–$4)	$17 ($15–$18)	$8 ($7–$8)	$218 ($197–$240)	$11 ($8–$13)
Same-day surgery	$32 ($30–$34)	$3 ($2–$3)	$35 ($32–$37)	$8 ($7–$8)	$170 ($156–$185)	$3 ($3–$3)
Inpatient hospitalization	$491 ($452–$530)	$36 ($30–$42)	$404 ($357–$451)	$60 ($55–$66)	$5451 ($5084–$5818)	$154 ($109–$198)
Medications	$63 ($58–$68)	$17 ($15–$18)	$65 ($58–$73)	$43 ($40–$46)	$247 ($231–$264)	$23 ($21–$25)
Home care	$86 ($79–$93)	$7 ($6–$8)	$61 ($54–$68)	$14 ($12–$16)	$467 ($440–$494)	$17 ($8–$27)
Continuing care	$5 ($1–$8)	$11 ($8–$14)	$4 (−$1–$8)	$22 ($17–$27)	$20 ($10–$29)	$23 ($14–$31)
Long-term care	$9 ($5–$14)	$20 ($16–$24)	$8 ($2–$14)	$32 ($25–$38)	$43 ($31–$55)	$42 ($32–$53)
Total cost	$1139 ($1079–$1199)	$122 ($110–$135)	$923 ($852–$995)	$254 ($232–$276)	$9004 ($8545–$9462)	$326 ($265–$387)

*EAC* esophageal adenocarcinoma, *CI* confidence intervals

^a^Mean health care costs are expressed in 2016 Canadian dollars per 30 patient-days

Estimates of the average total net costs of EAC care per 30 patient-days were highest in the terminal phase ($8678, 96% of overall EAC net costs), followed by the initial phase ($1016, 11%) and continuing care phase ($669, 7%) of overall EAC net costs (Table 3 and see Additional file 4: Figure S2a-S2d). The net costs of inpatient hospitalization (85–97% of the mean health care costs of inpatient hospitalization in Table 2) and outpatient visits (75–97% of the mean health care of outpatient visits in Table 2) due to EAC accounted for the highest cost categories across all three phases. We reported bootstrap mean and 95% CIs derived from the generalized estimating equations on Additional file 5: Table S4. With large sample sizes, the bootstrap samples results are similar to the original sample.

**Table 3:** Mean net costs^a^ of care due to esophageal adenocarcinoma according to service category and phase of care, 2003–2011

Service category	Overall	Initial Phase	Continuing Care Phase	Terminal Phase
	Mean (95% CI)	Mean (95% CI)	Mean (95% CI)	Mean (95% CI)
N	6022	4227	3521	6022
Outpatient visits	$1279 ($1215–$1343)	$155 ($148–$162)	$117 ($109–$125)	$1226 ($1161–$1291)
Emergency department visits	$209 ($188–$231)	$17 ($16–$18)	$9 ($8–$11)	$208 ($186–$230)
Same-day surgery	$179 ($164–$193)	$29 ($27–$31)	$27 ($24–$30)	$167 ($153–$182)
Inpatient hospitalization	$5501 ($5135–$5867)	$455 ($415–$494)	$343 ($296–$390)	$5297 ($4929–$5665)
Medications	$207 ($190–$225)	$46 ($41–$51)	$22 ($14–$30)	$224 ($208–$240)
Home care	$479 ($450 –$509)	$79 ($72–$86)	$47 ($39–$55)	$449 ($421–$478)
Continuing care	-$32 (−$47– -$17)	-$7 (−$11– -$2)	-$18 (−$25– -$12)	-$3 (−$15–$9)
Long-term care	-$44 (−$64– -$24)	-$11 (−$17– -$4)	-$24 (−$33– -$15)	$0 (−$15–$16)
Total net costs	$9002 ($8547–$9456)	$1016 ($955–$1078)	$669 ($594–$743)	$8678 ($8217–$9139)

Net costs of care due to esophageal adenocarcinoma were generated using generalized estimating equations

*EAC* esophageal adenocarcinoma, *CI* confidence intervals

^a^Mean health care costs are expressed in 2016 Canadian dollars per 30 patient-days

### Total net costs of care by stage at diagnosis and treatment for EAC

Stage IV at EAC diagnosis accounted the highest total net costs per 30 patient-days and approximately 10% of the total costs in the initial phase ($1010; 95% CI, $887–$1134), 6% in the continuing care phase ($620; 95% CI, $461–$780), and 100% in the terminal phase ($10,000; 95% CI, $9106–$10,894). Stage 0-I at EAC diagnosis accounted the lowest total costs. For stage 0-I, 16% of the total costs in the initial phase ($804; 95% CI, $626–$982), 13% in the continuing care phase ($646; 95% CI, $481–$810), and 83% in the terminal phase ($4249; 95% CI, $1789–$6710) (Table 4 and see Additional file 6: Figure S3a-S3d).

**Table 4:** Overall and phase of care net cost of health care resources by stage at diagnosis and treatment type for the esophageal adenocarcinoma cohort, 2003–2011

Cost category	Overall		Initial Phase		Continuing Care Phase		Terminal Phase
	Mean^a^ (95% CI)	N (Cases)	Mean^a^ (95% CI)	N (Cases)	Mean^a^ (95% CI)	N (Cases)	Mean^a^ (95% CI)
Stage at EAC diagnosis							
Stage 0-I	$5094 ($2659–$7529)	102	$804 ($626–$982)	64	$646 ($481–$810)	126	$4249 ($1789–$6710)
Stage II	$6192 ($5368–$7015)	282	$999 ($887–$1111)	161	$696 ($600–$791)	416	$5426 ($4574–$6277)
Stage III	$7413 ($6679–$8147)	273	$1254 ($1142–$1366)	136	$708 ($565–$851)	451	$6652 ($5879–$7425)
Stage IV	$9978 ($9093–$10,864)	222	$1010 ($887–$1134)	74	$620 ($461–$780)	930	$10,000 ($9106–$10,894)
Type of EAC treatment							
Surgery alone	$7785 ($6667–$8903)	361	$996 ($856–$1135)	215	$868 ($690–$1046)	533	$6937 ($5802–$8071)
Chemotherapy alone	$8607 ($7565–$9650)	161	$1109 ($935–$1282)	86	$590 ($466–$715)	338	$8168 ($7091–$9245)
Radiotherapy alone	$7998 ($7095–$8901)	228	$1330 ($1187–$1474)	118	$630 ($518–$742)	405	$7285 ($6344–$8225)
Surgery + chemotherapy	$5801 ($4751–$6850)	81	$1089 ($862–$1316)	52	$878 ($634–$1123)	118	$4832 ($3710–$5954)
Surgery + radiotherapy	$12,417 ($2067–$22,767)	–	$1080 ($494–$1667)	–	$170 (−$200–$539)	6	$12,237 ($1541–$22,933)
Chemotherapy + radiotherapy	$7671 ($6023–$9320)	68	$1129 ($909–$1350)	40	$846 ($650–$1042)	109	$6849 ($5107–$8590)
Surgery + chemotherapy + radiotherapy	$4743 ($1755–$7731)	7	$1323 ($757–$1890)	–	$236 ($111–$362)	7	$3519 ($684–$6354)
No treatment	$10,152 ($9431–$10,873)	377	$765 ($688–842)	136	$318 ($243–$393)	1495	$10,238 ($9512–$10,965)

^a^Mean health care costs are expressed in 2016 Canadian dollars per 30 patient-days. ‘–’, counts less than six are suppressed

*EAC* esophageal adenocarcinoma, *CI* confidence intervals

The mean net costs per 30 patient-days of patients receiving radiotherapy alone was highest in the initial phase ($1330; 95% CI, $1187–$1474) followed by surgery plus chemotherapy plus radiotherapy ($1323; 95% CI, $757–$1890), chemotherapy plus radiotherapy ($1129; 95% CI, ($909–$1350), chemotherapy alone ($1109; 95% CI, $935–$1282), surgery plus chemotherapy ($1089; 95% CI, $862–$1316), surgery plus radiotherapy ($1080; 95% CI, $494–$1667), and surgery alone ($996; 95% CI, $856–$1135). The mean costs of patients receiving surgery plus chemotherapy ($878; 95% CI, $634–$1123) were highest in the continuing care phase followed by surgery alone ($868; 95% CI, $690–$1046) and chemotherapy plus radiotherapy ($846; 95% CI, $650–$1042). The mean costs of patients that received surgery plus radiotherapy were highest in the terminal phase ($12,237; 95% CI, $1541–$22,933) followed by those not receiving treatment ($10,238; 95% CI, $9512–$10,965) and those receiving chemotherapy alone ($8168; 95% CI, $7091–$9245) (Table 4 and see Additional file 7: Figure S4a-S4d).

### Predictors of total net costs of care in individuals diagnosed with EAC

Univariate and multivariate predictors of total net costs of care in individuals diagnosed with EAC are summarized in Tables 5 and 6. Several patient characteristics were significant predictors of total net costs of care per 30 patient-days. In the initial phase, predictors of higher costs associated with EAC included all stages at EAC diagnosis compared with stage 0-I (*P* < 0.001), all treatments for EAC except surgery plus radiotherapy compared with no treatment (*P* < 0.001), and year of EAC diagnosis from 2006 to 2011 compared with 2003 (*P* < 0.001) (Table 6). The multivariate coefficients for stage indicate in the initial phase, stage III compared to stage 0–1 cost $1.51 more per 30 patient-days controlling for other factors. Patients diagnosed with EAC on 2011 compared to 2003 cost $2.41 more per 30 patient-days, after controlling for other factors. Patients who received surgery plus chemotherapy plus radiotherapy were associated with $2.57 increase in cost per 30 patient-days compared to the no treatment group controlling for all other factors.

**Table 5:** Predictors of total net costs of care in individuals with a diagnosis of esophageal adenocarcinoma according to phase of care, 2003–2011: Univariate generalized estimation equations

Variable	Initial Phase			Continuing Care Phase			Terminal Phase		
	Estimate	95% CI	*P*-value	Estimate	95% CI	*P*-value	Estimate	95% CI	*P*-value
	*N* = 4178			*N* = 3472			*N* = 5972		
Age group at index date (years)^*^									
< 50	Reference			Reference			Reference		
50–54	0.112	− 0.121–0.343	0.343	0.303	0.087–0.518	0.006	−0.038	− 0.272–0.194	0.749
55–59	− 0.011	− 0.236–0.211	0.921	0.104	− 0.106–0.311	0.328	− 0.106	− 0.331–0.117	0.355
60–64	0.022	− 0.191–0.230	0.838	0.340	0.141–0.534	0.001	0.008	− 0.205–0.216	0.942
65–69	0.162	− 0.053–0.374	0.135	0.549	0.349–0.746	< 0.001	0.006	−0.210–0.217	0.960
70–74	0.156	−0.063–0.372	0.158	0.751	0.548–0.951	< 0.001	0.246	0.030–0.458	0.024
75–79	− 0.041	− 0.264–0.179	0.716	0.786	0.578–0.990	< 0.001	0.220	0.001–0.435	0.047
80–84	0.184	− 0.059–0.426	0.137	0.854	0.624–1.085	< 0.001	0.238	0.004–0.470	0.045
≥ 85	0.273	0.003–0.549	0.049	0.992	0.730–1.260	< 0.001	0.328	0.076–0.581	0.011
Gender									
Male	Reference			Reference			Reference		
Female	0.006	−0.125–0.141	0.928	0.084	− 0.041–0.213	0.193	0.041	− 0.087–0.172	0.539
Residence									
Urban	Reference			Reference			Reference		
Rural	−0.006	− 0.133–0.124	0.924	− 0.073	− 0.194–0.052	0.246	0.001	− 0.124–0.129	0.987
Birth country									
Outside of Canada	Reference			Reference			Reference		
Canada	0.006	− 0.130–0.139	0.932	0.067	−0.173–0.298	0.574	0.020	−0.073–0.112	0.668
Income quintile^†^									
Q1 (lowest)	− 0.040	− 0.196–0.116	0.619	0.017	− 0.131–0.164	0.822	0.141	− 0.013–0.295	0.072
Q2	−0.077	− 0.234–0.079	0.331	− 0.023	− 0.172–0.125	0.758	0.213	0.059–0.366	0.007
Q3	−0.091	− 0.250–0.068	0.260	− 0.067	− 0.217–0.084	0.383	0.247	0.090–0.404	0.002
Q4	0.043	−0.111–0.196	0.587	0.151	0.005–0.296	0.042	0.117	−0.037–0.27	0.135
Q5 (highest)	Reference			Reference			Reference		
Ontario health region^**^									
Central	Reference			Reference			Reference		
Erie St. Clair	−0.073	−0.353–0.212	0.610	0.061	−0.200–0.327	0.649	0.052	−0.227–0.335	0.716
South West	−0.071	− 0.316–0.174	0.571	− 0.326	− 0.557– − 0.097	0.005	0.173	− 0.069–0.412	0.159
Waterloo Wellington	0.085	− 0.186–0.360	0.539	−0.094	− 0.351–0.166	0.475	0.214	− 0.055–0.486	0.121
Hamilton Niagara Haldimand Brant	0.048	−0.174–0.265	0.666	0.011	−0.198–0.216	0.917	0.062	−0.160–0.279	0.579
Central West	0.383	0.068–0.712	0.020	0.326	0.031–0.633	0.033	0.144	−0.175–0.476	0.386
Mississauga	0.018	−0.266–0.309	0.901	−0.090	− 0.358–0.184	0.513	0.235	− 0.048–0.523	0.106
Toronto Central	0.046	−0.214–0.308	0.729	0.328	0.082–0.576	0.009	0.291	0.032–0.551	0.028
Central East	−0.035	− 0.267–0.194	0.767	−0.060	− 0.278–0.155	0.586	0.053	− 0.179–0.281	0.651
South East	0.051	−0.206–0.309	0.696	0.049	−0.193–0.292	0.692	−0.085	− 0.342–0.172	0.515
Champlain	0.047	−0.182–0.272	0.686	0.043	−0.173–0.256	0.691	0.032	−0.199–0.260	0.782
North Simcoe Muskoka	−0.025	− 0.304–0.259	0.861	− 0.014	− 0.276–0.254	0.920	0.026	− 0.252–0.309	0.855
North East	− 0.046	− 0.308–0.218	0.732	− 0.090	− 0.337–0.158	0.474	0.245	− 0.017–0.507	0.067
North West	0.010	− 0.338–0.379	0.957	− 0.026	− 0.359–0.329	0.884	0.164	− 0.178–0.525	0.359
ADGs^‡^									
0	Reference			Reference			Reference		
1–3	0.201	−1.302–1.236	0.747	0.642	−0.719–1.609	0.263	−0.341	−1.393–0.470	0.463
4–7	0.083	−1.407–1.087	0.893	0.801	−0.547–1.738	0.155	−0.564	− 1.595–0.212	0.212
8–10	0.211	− 1.277–1.213	0.729	1.045	− 0.301–1.980	0.063	− 0.507	− 1.537–0.265	0.259
11+	0.334	− 1.153–1.334	0.584	1.504	0.159–2.436	0.008	−0.472	−1.501–0.297	0.293
Stage at EAC diagnosis^§^									
Stage 0-I	Reference			Reference			Reference		
Stage II	0.191	0.006–0.371	0.040	0.054	− 0.164–0.266	0.621	0.229	0.014–0.435	0.033
Stage III	0.396	0.210–0.577	< 0.001	0.067	− 0.157–0.284	0.551	0.422	0.209–0.627	< 0.001
Stage IV	0.201	0.010–0.388	0.037	−0.028	− 0.277–0.219	0.823	0.814	0.613–1.006	< 0.001
EAC treatment^¶^									
No treatment	Reference			Reference			Reference		
Surgery alone	0.231	0.106–0.356	< 0.001	0.673	0.512–0.832	< 0.001	−0.375	− 0.485– − 0.263	< 0.001
Chemotherapy alone	0.327	0.170–0.489	< 0.001	0.389	0.189–0.592	< 0.001	− 0.218	− 0.349– − 0.084	0.001
Radiotherapy alone	0.493	0.352–0.636	< 0.001	0.435	0.251–0.619	< 0.001	−0.328	− 0.450– − 0.203	< 0.001
Surgery + chemotherapy	0.312	0.108–0.524	0.003	0.682	0.447–0.925	< 0.001	− 0.717	− 0.921– − 0.500	< 0.001
Surgery + radiotherapy	0.304	− 0.700–1.799	0.620	− 0.300	−1.193–0.953	0.572	0.173	− 0.610–1.228	0.705
Chemotherapy + radiotherapy	0.344	0.126–0.574	0.003	0.653	0.396–0.923	< 0.001	−0.387	− 0.598– − 0.162	0.001
Surgery + chemotherapy + radiotherapy	0.488	− 0.098–1.210	0.139	− 0.154	−1.047–1.098	0.771	−1.011	− 1.743– − 0.046	0.017
Year of EAC diagnosis^€^									
2003	Reference			Reference			Reference		
2004	0.015	−0.208–0.238	0.896	0.010	−0.193–0.213	0.922	−0.131	− 0.352–0.090	0.245
2005	−0.056	− 0.285–0.174	0.633	− 0.158	− 0.365–0.049	0.134	0.064	− 0.159–0.287	0.573
2006	0.401	0.183–0.619	< 0.001	0.073	− 0.129–0.273	0.478	0.033	− 0.183–0.248	0.763
2007	0.292	0.069–0.514	0.010	−0.053	− 0.257–0.150	0.607	0.175	− 0.044–0.394	0.117
2008	0.367	0.151–0.581	0.001	−0.181	− 0.377–0.014	0.069	0.351	0.139–0.561	0.001
2009	0.527	0.316–0.735	< 0.001	−0.274	− 0.467– − 0.084	0.005	0.327	0.119–0.532	0.002
2010	0.462	0.253–0.668	< 0.001	−0.729	− 0.922– −0.539	< 0.001	0.310	0.103–0.514	0.003
2011	0.541	0.333–0.746	< 0.001	−1.723	− 1.916– − 1.531	< 0.001	0.222	0.016–0.425	0.033

Initial Phase, overall *P* values (unadjusted): ^*^age group at index date: *P* = 0.084; ^†^income quintile: *P* = 0.405; ^**^Ontario health region: *P* = 0.541; ^‡^ADGs: *P* < 0.005; ^§^stage at EAC diagnosis: *P* < 0.001; ^¶^treatment for EAC: *P* < 0.001; ^€^year of EAC diagnosis: *P* < 0.001

Continuing Care Phase, overall *P* values (unadjusted): ^*^age group at index date: *P* < 0.001; ^†^income quintile: *P* = 0.038; ^**^Ontario health region: *P* < 0.001; ^‡^ADGs: *P* < 0.001; ^§^stage at EAC diagnosis: *P* = 0.794; ^¶^treatment for EAC: *P* < 0.001; ^€^year of EAC diagnosis: *P* < 0.001

Terminal Phase, overall *P* values (unadjusted): ^*^age group at index date: *P* < 0.001; ^†^income quintile: *P* = 0.022; ^**^Ontario health region: *P* = 0.176; ^‡^ADGs: *P* = 0.304; ^§^stage at EAC diagnosis: *P* < 0.001; ^¶^treatment for EAC: *P* < 0.001; ^€^year of EAC diagnosis: *P* < 0.001

**Table 6:** Predictors of total net costs of care in individuals with a diagnosis of esophageal adenocarcinoma according to phase of care, 2003–2011: Multivariate generalized estimation equations with a log link function and gamma distribution

Variable	Initial Phase			Continuing Care Phase		Terminal Phase			
	Estimate	95% CI	*P*-value	Estimate	95% CI	*P*-value	Estimate	95% CI	*P*-value
Intercept	6.012	5.588–6.461	< 0.001	4.402	3.653–5.338	< 0.001	7.718	7.176–8.288	< 0.001
Age group at index date (years)^*^									
< 50	Reference						Reference		
50–54	− 0.021	− 0.243–0.200	0.853				−0.024	− 0.239–0.189	0.823
55–59	− 0.102	− 0.321–0.114	0.355				−0.175	− 0.385–0.033	0.099
60–64	− 0.111	− 0.317–0.092	0.289				−0.004	− 0.203–0.193	0.971
65–69	0.085	− 0.127–0.295	0.428				−0.100	− 0.302–0.100	0.331
70–74	0.064	−0.159–0.286	0.572				0.276	0.068–0.483	0.009
75–79	−0.184	− 0.409–0.041	0.109				0.125	−0.086–0.334	0.242
80–84	− 0.049	− 0.315–0.221	0.718				0.040	−0.190–0.271	0.732
≥ 85	−0.377	− 0.703– − 0.036	0.027				0.181	− 0.113–0.484	0.235
Residence									
Urban	Reference								
Rural	−0.159	−0.293– − 0.022	0.022						
Income quintile^†^									
Q1 (lowest)	0.156	−0.007–0.320	0.061						
Q2	−0.074	−0.239–0.091	0.378						
Q3	−0.069	− 0.232–0.094	0.406						
Q4	−0.112	− 0.269–0.045	0.162						
Q5 (highest)	Reference								
Ontario health region^**^									
Central							Reference		
Erie St. Clair				0.107	−0.180–0.401	0.468	0.137	− 0.148–0.425	0.348
South West				−0.181	− 0.460–0.102	0.205	0.151	− 0.098–0.397	0.233
Waterloo Wellington				−0.029	− 0.326–0.277	0.851	0.356	0.089–0.623	0.009
Hamilton Niagara Haldimand Brant				0.026	−0.212–0.262	0.829	0.101	− 0.122–0.317	0.369
Central West				0.169	−0.137–0.485	0.284	−0.125	− 0.471–0.238	0.488
Mississauga				0.103	−0.216–0.433	0.534	0.213	−0.118–0.558	0.216
Toronto Central				0.122	−0.160–0.409	0.401	0.197	−0.073–0.469	0.154
Central East				0.076	−0.165–0.315	0.537	−0.080	− 0.320–0.158	0.513
South East				−0.227	− 0.491–0.039	0.093	− 0.067	− 0.319–0.182	0.598
Champlain				0.200	−0.035–0.432	0.093	0.117	−0.117–0.346	0.322
North Simcoe Muskoka				−0.047	− 0.333–0.245	0.747	0.030	− 0.250–0.314	0.833
North East				−0.103	− 0.387–0.188	0.483	0.396	0.124–0.670	0.004
North West				−0.537	− 0.899– − 0.152	0.005	0.405	0.086–0.734	0.014
ADGs^‡^									
0				Reference					
1–3				1.619	0.659–2.408	< 0.001			
4–7				1.636	0.707–2.373	< 0.001			
8–10				1.712	0.788–2.442	< 0.001			
11+				1.897	0.975–2.623	< 0.001			
Stage at EAC diagnosis^§^									
Stage 0-I	Reference						Reference		
Stage II	0.220	0.043–0.392	0.014				0.085	−1.167–1.938	0.911
Stage III	0.414	0.236–0.587	< 0.001				0.900	0.461–1.315	< 0.001
Stage IV	0.325	0.135–0.511	0.001				1.697	0.640–3.143	0.006
EAC treatment^¶^									
No treatment	Reference			Reference			Reference		
Surgery alone	0.237	0.087–0.386	0.002	0.658	0.500–0.815	< 0.001	−4.123	−6.577– −0.977	0.001
Chemotherapy alone	0.190	0.012–0.371	0.038	0.420	0.223–0.620	< 0.001	−1.309	−3.154–0.547	0.150
Radiotherapy alone	0.407	0.250–0.565	< 0.001	0.513	0.334–0.693	< 0.001	−0.841	−2.750–1.280	0.387
Surgery + chemotherapy	0.423	0.185–0.671	0.001	0.590	0.362–0.824	< 0.001	−1.290	−2.019– −0.464	0.001
Surgery + radiotherapy	0.733	−0.491–2.695	0.338	−0.510	−1.375–0.653	0.312	− 0.038	− 1.564–2.854	0.970
Chemotherapy + radiotherapy	0.449	0.209–0.699	< 0.001	0.617	0.368–0.876	< 0.001	−1.292	−3.592–1.863	0.310
Surgery + chemotherapy + radiotherapy	0.942	0.243–1.810	0.017	−0.104	− 0.974–1.061	0.837	− 0.808	−2.210–1.287	0.344
Year of EAC diagnosis^€^									
2003	Reference			Reference			Reference		
2004	0.184	− 0.212–0.555	0.346	0.012	− 0.236–0.258	0.924	−0.085	− 0.459–0.267	0.647
2005	0.357	−0.044–0.734	0.071	0.095	−0.154–0.343	0.454	0.420	0.050–0.768	0.022
2006	0.615	0.229–0.973	0.001	0.355	0.108–0.600	0.005	0.342	− 0.020–0.679	0.054
2007	0.379	−0.010–0.742	0.047	0.276	0.026–0.525	0.030	0.473	0.108–0.814	0.009
2008	0.653	0.271–1.007	0.001	0.311	0.071–0.547	0.010	0.561	0.202–0.894	0.001
2009	0.815	0.433–1.168	< 0.001	0.224	−0.015–0.460	0.064	0.660	0.303–0.991	< 0.001
2010	0.675	0.294–1.026	< 0.001	−0.099	− 0.339–0.139	0.417	0.537	0.180–0.868	0.002
2011	0.878	0.495–1.231	< 0.001	−0.458	− 0.735– − 0.178	0.001	0.531	0.169–0.869	0.003
Income quintile-Comorbidity interaction^††^									
Q1 (lowest)*ADGs							−0.036	− 0.184–0.112	0.629
Q2*ADGs							−0.096	− 0.165– − 0.027	0.006
Q3*ADGs							0.017	−0.041–0.077	0.563
Q4*ADGs							−0.063	−0.111– − 0.016	0.009
Comorbidity-EAC stage interaction^‡‡^									
ADGs 1–3*EAC stage II							1.132	−0.820–2.588	0.173
ADGs 1–3*EAC stage III							−0.446	−1.181–0.319	0.242
ADGs 1–3*EAC stage IV							−0.733	−2.219–0.399	0.256
ADGs 4–7*EAC stage II							0.274	− 1.578–1.519	0.715
ADGs 4–7*EAC stage III							−0.090	−0.482–0.304	0.652
ADGs 4–7*EAC stage IV							−0.479	−1.903–0.530	0.424
ADGs 8–10*EAC stage II							0.274	−1.569–1.500	0.712
ADGs 8–10*EAC stage III							−0.269	−0.610–0.072	0.122
ADGs 8–10*EAC stage IV							−0.821	−2.239–0.176	0.168
ADGs 11 + *EAC stage II							0.345	−1.490–1.554	0.639
ADGs 11 + *EAC stage IV							−0.535	−1.950–0.456	0.367
Comorbidity-EAC treatment interaction^‡‡‡^									
ADGs 1–3*Surgery alone							4.145	0.983–6.626	0.001
ADGs 1–3*Chemotherapy alone							0.885	−1.062–2.881	0.364
ADGs 1–3*Radiotherapy alone							1.126	−1.021–3.076	0.256
ADGs 1–3*Surgery + chemotherapy							−1.279	−3.018–1.679	0.238
ADGs 1–3*Chemotherapy + radiotherapy							1.926	−1.213–4.247	0.128
ADGs 4–7*Surgery alone							3.650	0.514–6.087	0.004
ADGs 4–7*Chemotherapy alone							1.304	−0.444–3.054	0.126
ADGs 4–7*Radiotherapy alone							1.135	− 0.936–2.983	0.227
ADGs 4–7*Surgery + chemotherapy							−0.003	− 0.684–0.729	0.993
ADGs 4–7*Surgery + radiotherapy							−1.133	−4.416–2.149	0.433
ADGs 4–7*Chemotherapy + radiotherapy							1.982	−1.091–4.148	0.100
ADGs 4–7*Surgery + chemotherapy + radiotherapy							0.274	−1.907–2.458	0.792
ADGs 8–10*Surgery alone							3.884	0.754–6.313	0.002
ADGs 8–10*Chemotherapy alone							1.419	−0.318–3.156	0.093
ADGs 8–10*Radiotherapy alone							0.784	−1.282–2.625	0.402
ADGs 8–10*Surgery + chemotherapy							0.319	−0.281–0.943	0.304
ADGs 8–10*Surgery + radiotherapy							0.135	−3.125–3.394	0.925
ADGs 8–10*Chemotherapy + radiotherapy							2.654	−0.410–4.800	0.027
ADGs 11 + *Surgery alone							4.185	1.060–6.604	0.001
ADGs 11 + *Chemotherapy alone							1.225	−0.499–2.950	0.143
ADGs 11 + *Radiotherapy alone							0.986	−1.076–2.823	0.291
ADGs 11 + *Chemotherapy + radiotherapy							2.253	−0.808–4.395	0.059
EAC stage-EAC treatment interaction^§§^									
EAC stage II*Surgery alone							0.107	−0.446–0.668	0.706
EAC stage II*Chemotherapy alone							0.031	−0.819–0.814	0.940
EAC stage II*Radiotherapy alone							−0.161	−0.814–0.476	0.622
EAC stage II*Surgery + chemotherapy							0.667	−0.294–1.576	0.158
EAC stage II*Chemotherapy + radiotherapy							−1.105	−2.191– −0.146	0.031
EAC stage III*Surgery alone							−0.182	−0.725–0.368	0.513
EAC stage III*Chemotherapy alone							−0.310	−1.158–0.470	0.452
EAC stage III*Radiotherapy alone							−0.215	− 0.861–0.415	0.506
EAC stage III*Surgery + chemotherapy							1.146	0.194–2.038	0.014
EAC stage III*Chemotherapy + radiotherapy							−0.804	−1.894–0.158	0.119
EAC stage III*Surgery + chemotherapy + radiotherapy							0.397	−1.813–2.610	0.707
EAC stage IV*Surgery alone							−0.091	−0.664–0.497	0.759
EAC stage IV*Chemotherapy alone							−0.115	−0.921–0.612	0.767
EAC stage IV*Radiotherapy alone							−0.135	− 0.757–0.471	0.666
EAC stage IV*Surgery + chemotherapy							1.315	0.326–2.272	0.008
EAC stage IV*Chemotherapy + radiotherapy							−1.162	−2.233– −0.224	0.021

*CI* confidence interval, *ADGs* Aggregated Diagnosis Groups, *EAC* esophageal adenocarcinoma

Initial Phase: final multivariable analysis adjusted for age group at index date, residence, income quintile, stage at EAC diagnosis. Treatment for EAC, and year of EAC diagnosis (index date). Residence and income quintile variables that were non-significant in the univariate analysis were added in the final model and became significant. Overall *P* values (adjusted): ^*^age group at index date: *P* = 0.045; ^†^income quintile: *P* = 0.009; ^§^stage at EAC diagnosis: *P* < 0.001; ^¶^treatment for EAC: *P* < 0.001; ^€^year of EAC diagnosis: *P* < 0.001

Continuing Care Phase: final multivariable analysis adjusted for Ontario health region, comorbidity, measured by the Johns Hopkins ADGs, treatment for EAC, and year of EAC diagnosis (index date). Overall *P* values (adjusted): ^**^Ontario health region: *P* = 0.008; ^‡^comorbidity: *P* < 0.001; ^¶^treatment for EAC: *P* < 0.001; ^€^year of EAC diagnosis: *P* < 0.001

Terminal Phase: final multivariable analysis adjusted for age group at index date, Ontario health region, stage at EAC diagnosis, treatment for EAC, year of EAC diagnosis (index date), income quintile and comorbidity interaction, comorbidity and stage at EAC diagnosis interaction, comorbidity and EAC treatment interaction, and stage at EAC diagnosis and treatment for EAC interaction. Overall *P* values (adjusted): ^*^age group at index date: *P* < 0.001; ^**^Ontario health region: *P* < 0.001; ^§^stage at EAC diagnosis: *P* < 0.001; ^¶^treatment for EAC: *P* = 0.025; ^€^year of EAC diagnosis: *P* < 0.001; ^††^Income quintile-Comorbidity interaction: *P* = 0.006; ^‡‡^Comorbidity-EAC stage interaction: *P* = 0.009; ^‡‡‡^Comorbidity- EAC treatment interaction: *P* = 0.010; ^§§^EAC stage-EAC treatment interaction: *P* = 0.002

In the continuing care phase, predictors of higher cost associated with EAC included comorbidity measured by the Johns Hopkins ADGs (from ADGs 1–3 to ADGs 11+ compared with no comorbidity, *P* < 0.001) and all treatments for EAC except surgery plus radiotherapy and surgery plus chemotherapy plus radiotherapy (*P* < 0.001), and year of EAC diagnosis from 2006 to 2008 (*P* < 0.05).

In the terminal phase, predictors of higher cost associated with EAC included 70–74 years of age at index date (*P* = 0.009), Ontario health region (Waterloo Wellington, *P* = 0.009; North East, *P* = 0.004; and North West, *P* = 0.014), stage III (*P* < 0.001) and stage IV (*P* = 0.006) at EAC diagnosis, year of EAC diagnosis from 2005 to 2011 (except 2006; *P* < 0.001), comorbidity (ADGs 1–3, 4–7, 8–10 and 11+) and surgery alone interaction (*P* = 0.001, *P* = 0.004, *P* = 0.002 and *P* = 0.001, respectively), comorbidity (ADGs 8–10) and chemotherapy plus radiotherapy interaction (*P* = 0.027) and EAC stage-EAC surgery plus chemotherapy interactions (stage III, *P* = 0.014) and stage IV (*P* = 0.008) (Table 6). In the terminal phase, patients in stage IV were associated with $5.46 increase in cost per 30 patient-days compared to those in stage 0–1 controlling for other factors.

#### Sensitivity analysis

In the sensitivity analysis assigning 6 months after the diagnosis to the initial phase and 6 months preceding death to the terminal phase, there was a significant increase (85%) in the total net costs of care in the continuing care phase and a modest increase (42%) in the initial phase compared with the total net costs of care in the primary analysis (Additional file 8: Table S5). The mean net costs of surgery plus radiotherapy (108%) and all treatments significantly increased in the initial phase (23–108%) and continuing care phase (63–639%), respectively compared with the primary analysis (Additional file 8: Table S6). Predictors of total net costs of care associated with EAC were similar to the primary analysis in the terminal phase (Additional file 8: Table S7).

## Discussion

This population-based retrospective cohort study examined phase-specific net costs of care per 30 patient-days attributable to EAC from a public payer perspective, total net costs of care by stage and treatment for EAC, and predictors of total net costs of care in individuals diagnosed with EAC by phase of care. The aggregated total net health care costs of EAC care were highest in the terminal phase, next highest in the initial phase, and the lowest cost was in the continuing care phase. Inpatient hospitalizations accounted for the largest share of costs in all phases, followed by outpatient visits and home care. Overall, stage IV at diagnosis and surgery plus radiotherapy for EAC accounted for the highest cost, in particular in the terminal phase. The factors that were associated with higher net costs of care included treatment for EAC, especially chemotherapy plus radiotherapy, surgery plus chemotherapy, radiotherapy alone, surgery alone, and chemotherapy alone in the initial and continuing care phases; intermediate or advanced stage and the latest year of EAC diagnosis in the initial and terminal phases; comorbidity in the continuing care phase; and older age at diagnosis (70–74 years) and Ontario health region (Waterloo Wellington, North East, North West) in the terminal phase. Associations like older age and lower income quintile may reflect medical factors such as comorbidity, or social factors like lesser social support that could lead to higher use of medical services. Finally, lower costs were associated with individuals diagnosed with EAC included 85 years of age and older at index date and rural residence in the initial phase.

Phase-specific costs are useful for estimating incidence-based and long-term care costs, defined as cumulative costs from the date of diagnosis to death [22]. In addition, phase-specific cost estimates constitute an important input for economic evaluation of prevention, screening, and treatment interventions [21, 22, 25]. Our phase-specific costing approach provided in-depth cost analysis to the specific net phase of care costs for EAC, compared to previous studies which only looked at overall costs. Recent and past studies analyzing hospital costs after complex esophageal surgical procedures indicate that postoperative complications are associated with increased resource utilization and costs [59, 60]. Such complications were captured in the phased costing approach we used. According to a large randomized trial, preoperative chemoradiotherapy is safe and leads to a significant increase in overall survival among patients with localized adenocarcinoma or squamous-cell carcinoma of the esophagus compared with those treated with surgery alone [61]. Esophageal cancer is often in an advanced stage when it is diagnosed, however. At later stages, esophageal cancer can be treated but not cured. The selection of prevention and treatment activities at different stages of disease can have significant impact on resource utilization [21, 62].

The strengths of our study include comprehensive cost estimation and rigorous propensity score matching between cases and controls, which was based on sociodemographic and comorbidity characteristics, providing unbiased estimates of the net costs of care. Our study results can inform publically funded health care systems on the cost of treatments for patients, considering stage and other sociodemographic and clinical patient characteristics. It can also aid detailed future planning of health care costs.

Our study has some limitations. Our cost estimates did not reflect the overall economic burden of EAC to the society. Because Ontario only provides comprehensive coverage for the elderly and those on social assistance, prescription medication costs were not included for patients < 65 years. However, 1722 (56.7%) of our patients are over age 65 years, and prescription drug costs only accounted for 10% of total costs for patients in this age group. Therefore, it is unlikely that the costs for prescription drug cost for patients under age 65 would significantly change our results. Additionally, since we matched patients on age, the missing prescription drug costs, which contributes towards the total costs, is also matched for cases and controls (patients who are matched and under 65 will not have this cost accounted for in the total compared to matched patients over the age of 65 which will have the cost included in total for cases and controls). No difference in results would be expected as the missing prescription is homogenously distributed between cases and controls. Although multiple imputation would provide a solution for missing data, in our case there is no readily available data for which to impute the cost of prescription drugs for patients under the age of 65. Prescription drug costs for patients over the age of 65 would not be valid to impute for patients under the age of 65. We therefore did not impute the value of prescription costs for patients with missing prescription drug costs. Moreover, a limitation of our study was that only overall cost was provided by type for patients. Individual health care costs with dates were not provided. Therefore, we were not able to investigate cost thresholds to determine cost phase boundaries and instead had to rely on previous research to determine cost phases. Furthermore, we estimated direct health care costs only and did not include patient out-of-pocket costs or loss of productivity, which are important elements of the cost of illness for society and individuals. Finally, we could not assess the effect of screening prior to Barrett’s esophagus or cancer diagnosis on costs. EAC patients with a prior Barrett’s esophagus diagnosis are commonly diagnosed with earlier stage disease and have improved survival compared with EAC patients with no prior Barrett’s esophagus diagnosis [63, 64].

## Conclusions

Our phase-specific longitudinal net costing approach for patients diagnosed with EAC until death identified three distinct cost phases, and found that costs were highest in the terminal phase, where inpatient hospitalization cost was the greatest contributor to total costs than in other phases. Furthermore, our findings suggest that the economic burden of EAC is significant and is expected to continue to increase due to the growth and aging of the population, the increase in the incidence of EAC, comorbidity, disease progression, and the potentially expensive treatments of the future. Further research is needed on methods to incorporate these phase-based costs into cost-effectiveness analyses for EAC treatment.

## Additional files

Additional file 1: Materials and Methods. **Table S1.** Fee codes used to define types of treatment for esophageal adenocarcinoma. **Table S2.** Codes used to define cases of esophageal adenocarcinoma. (DOCX 22 kb)

Additional file 2: **Figure S1.** Flowchart describing the selection of the study population. (JPG 90 kb)

Additional file 3: **Table S3.** Demographic characteristics of esophageal adenocarcinoma cases and controls, 2003–2011. (DOCX 21 kb)

Additional file 4: **Figure S2a-d.** Mean net costs of health care resources due to esophageal adenocarcinoma (difference between the mean costs for esophageal adenocarcinoma cases and for matched controls without cancer) according to phase of care, 2003–2011: (a) Overall; (b) Initial Phase; (c) Continuing Care Phase; and (d) Terminal Phase. (JPG 87 kb)

Additional file 5: **Table S4.** Bootstrap samples for mean net costs^*^ of care due to esophageal adenocarcinoma according to service category and phase of care, 2003–2011. (DOCX 13 kb)

Additional file 6: **Figure S3a-d.** Overall and phase of care net cost of health care resources by stage at diagnosis for esophageal adenocarcinoma, 2003–2011: (a) Overall; (b) Initial Phase; (c) Continuing Care Phase; and (d) Terminal Phase. (JPG 59 kb)

Additional file 7: **Figure S4a-d.** Overall and phase of care net cost of health care resources by treatment type for esophageal adenocarcinoma, 2003–2011: (a) Overall; (b) Initial Phase; (c) Continuing Care Phase; and (d) Terminal Phase. (JPG 80 kb)

Additional file 8: **Table S5.** Mean net costs^*^ of care due to esophageal adenocarcinoma according to service category and phase of care, 2003–2011: Sensitivity analysis. **Table S6.** Overall and phase of care net cost of health care resources by stage at diagnosis and treatment type for the esophageal adenocarcinoma cohort, 2003–2011: Sensitivity analysis. **Table S7.** Predictors of total net costs of care in individuals with a diagnosis of esophageal adenocarcinoma according to phase of care, 2003–2011: Multivariate generalized estimation equations with a log link function and gamma distribution – Sensitivity analysis. (DOCX 37 kb)

## Abbreviations

ADGs: Aggregated Diagnosis Groups
CI: Confidence interval
CIHI: Canadian Institute for Health Information
DAD: Discharge Abstract Database
EAC: Esophageal adenocarcinoma
GERD: Gastroesophageal reflux disease
ICD-9: International Statistical Classification of Diseases and Related Health Problems, 9th Revision
ICD-O-3: International Classification of Diseases for Oncology, Third Edition
LHIN: Local Health Integration Network
NACRS: National Ambulatory Care Reporting System
OCR: Ontario Cancer Registry
OHIP: Ontario Health Insurance Plan
