# Supplementary material for: Estimates and predictors of health care costs of esophageal adenocarcinoma: a population-based cohort study
Source: BMC Cancer. 2018 Jun 27;18:694. doi: 10.1186/s12885-018-4620-2 (PMC6020438; doi:10.1186/s12885-018-4620-2)
Supplement: Supplementary file 1 — Materials and Methods. Table S1. Fee codes used to define types of treatment for esophageal adenocarcinoma. Table S2. Codes used to define cases of esophageal adenocarcinoma. (DOCX 22 kb) [file 12885_2018_4620_MOESM1_ESM.docx]

**Table S1.** Fee codes used to define types of treatment for esophageal adenocarcinoma

| Treatment | Code Type | | References |
| --- | --- | --- | --- |
|  | OHIP | CIHI-DAD & NACRS |  |
| Surgical resection | S089, S090, S123, S125, S128 | 1NA55, 1NA76, 1NA77, 1NA87, 1NA88, 1NA89, 1NA90, 1NA91, 1NA92 | [[1](#_ENREF_1), [2](#_ENREF_2)] |
| Chemotherapy | G339, G345, G359, G281, G381, G382, G388 | 1NA35, 1ZZ35 | [[2](#_ENREF_2), [3](#_ENREF_3)] |
| Radiation therapy | X310, X311, X312, X313 | 1NA26, 1NA27 | [[2](#_ENREF_2), [4](#_ENREF_4)] |
| Palliative procedures |  |  | [[2](#_ENREF_2), [5](#_ENREF_5)] |
| Esophageal dilation | E696, E698, Z523, Z525, Z529 | 1NA50, 1NA53 |  |
| Drainage |  | 1NA52 |  |
| esophageal stenting | E629, S082, S083 |  |  |
| Laser debulking of tumor | E692, E695 | 1NA59 |  |
| Palliative care | E083, G511, G512, C882, C982, K023, C945 |  |  |

OHIP, Ontario Health Insurance Plan; CIHI, Canadian Institute for Health Information; DAD, Discharge Abstract Database; NACRS, National Ambulatory Care Reporting System

**Table S2.** Codes used to define cases of esophageal adenocarcinoma in Ontario Cancer Registry

| Data elements | ICD-9 | ICD-10 |
| --- | --- | --- |
| Esophageal adenocarcinoma | 150.0: Malignant neoplasm of cervical esophagus  150.1: Malignant neoplasm of thoracic esophagus  150.2: Malignant neoplasm of abdominal esophagus  150.3: Malignant neoplasm of upper third of esophagus  150.4: Malignant neoplasm of middle third of esophagus  150.5: Malignant neoplasm of lower third of esophagus  150.8: Malignant neoplasm of other specified part of esophagus  150.9: Malignant neoplasm of esophagus, unspecified site | C15.3: Malignant neoplasm of upper third of esophagus  C15.4: Malignant neoplasm of middle third of esophagus  C15.5: Malignant neoplasm of lower third of esophagus  C15.3: Malignant neoplasm of upper third of esophagus  C15.4: Malignant neoplasm of middle third of esophagus  C15.5: Malignant neoplasm of lower third of esophagus  C15.8: Malignant neoplasm of overlapping sites of esophagus  C15.9: Malignant neoplasm of esophagus, unspecified |
| Histology ICD-O-3 | 8140, 8141, 8143, 8144, 8145, 8147, 8200, 8201, 8255, 8260, 8261, 8262, 8263, 8430, 8480, 8481, 8560, 8562, 8570, 8571, 8572, 8573, 8574, 8575 | |

# ICD-9, International Statistical Classification of Diseases and Related Health Problems, 9th Revision; ICD-10, International Statistical Classification of Diseases and Related Health Problems 10th Revision (http://www.icd9data.com/2013/Volume1/140-239/150-159/150/default.htm); ICD-O-3, International Classification of Diseases for Oncology, Third Edition.

**References**

1. Digestive System Surgical Procedures. Available from: <http://www.health.gov.on.ca/en/pro/programs/ohip/sob/physserv/s_digest.pdf>. Accessed 9 March 2017.

2. Canadian Classification of Health Interventions. Volume Four — Alphabetical Index. Canadian Institute for Health Information 2012. Available from: https://[www.cihi.ca/en/cci_volume_four_2012_en.pdf](http://www.cihi.ca/en/cci_volume_four_2012_en.pdf). Accessed 9 March 2017.

3. Diagnostic and Therapeutic Procedures. Available from: <http://www.health.gov.on.ca/en/pro/programs/ohip/sob/physserv/j_diagth.pdf>. Accessed 9 March 2017.

4. Radiation Oncology. Available from: <http://health.gov.on.ca/en/pro/programs/ohip/sob/physserv/c_radiat.pdf>. Accessed 9 March 2017.

5. Guide to OHIP Billing for Palliative Care Services. Last Updated: February 23, 2015. Available from: https://hqic.ca/Pages/HqicPrivacyDocs.aspx?docNm=palliative-care-billing.pdf. Accessed 9 March 2017.
