# Supplementary figures and images for: Estimates and predictors of health care costs of esophageal adenocarcinoma: a population-based cohort study
Source: BMC Cancer. 2018 Jun 27;18:694. doi: 10.1186/s12885-018-4620-2 (PMC6020438; doi:10.1186/s12885-018-4620-2)

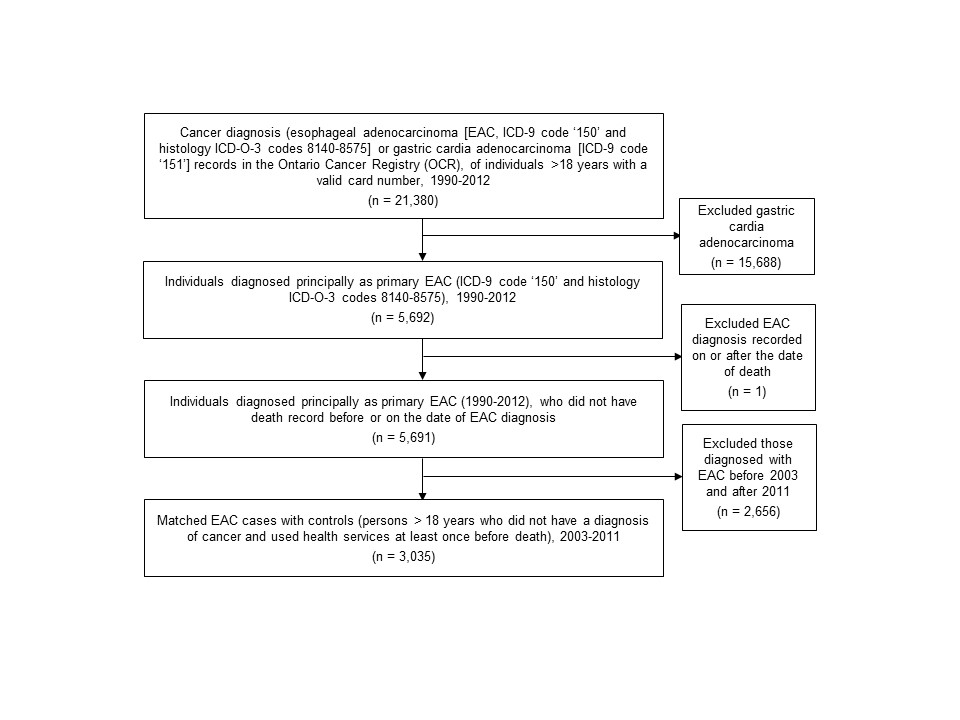

Supplement: Supplementary file 2 — Figure S1. Flowchart describing the selection of the study population. (JPG 90 kb) [file 12885_2018_4620_MOESM2_ESM.jpg]

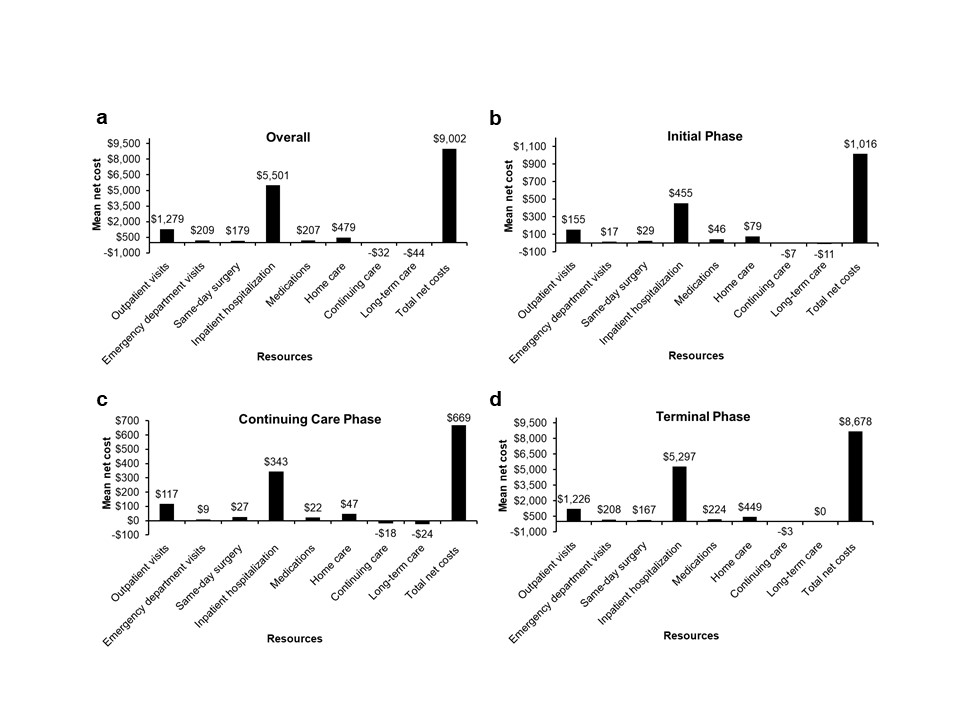

Supplement: Supplementary file 4 — Figure S2a-d. Mean net costs of health care resources due to esophageal adenocarcinoma (difference between the mean costs for esophageal adenocarcinoma cases and for matched controls without cancer) according to phase of care, 2003–2011: (a) Overall; (b) Initial Phase; (c) Continuing Care Phase; and (d) Terminal Phase. (JPG 87 kb) [file 12885_2018_4620_MOESM4_ESM.jpg]

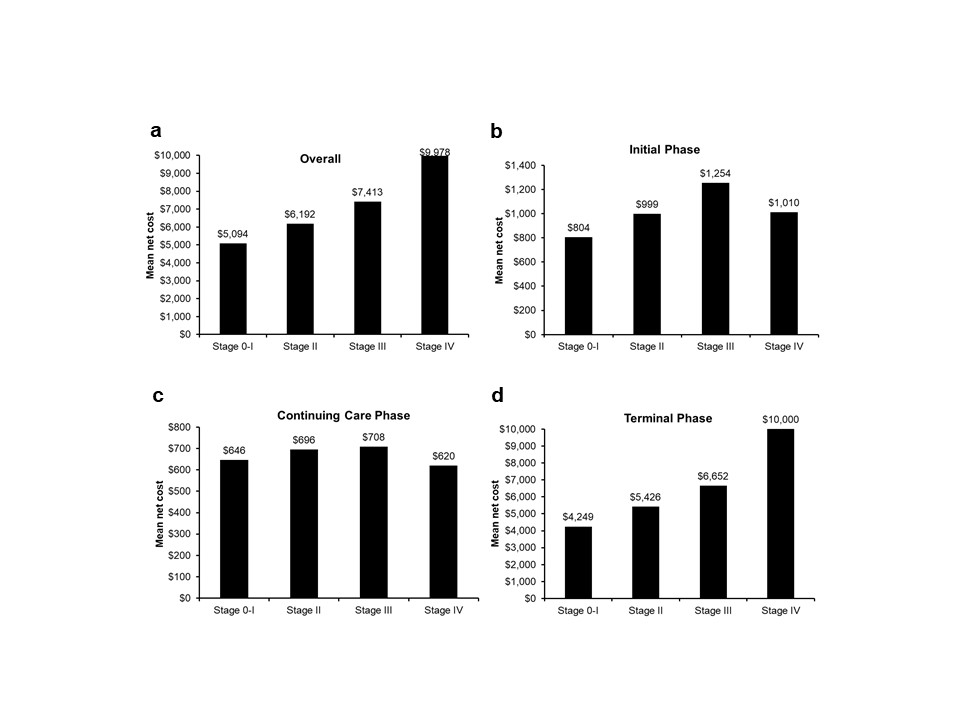

Supplement: Supplementary file 6 — Figure S3a-d. Overall and phase of care net cost of health care resources by stage at diagnosis for esophageal adenocarcinoma, 2003–2011: (a) Overall; (b) Initial Phase; (c) Continuing Care Phase; and (d) Terminal Phase. (JPG 59 kb) [file 12885_2018_4620_MOESM6_ESM.jpg]

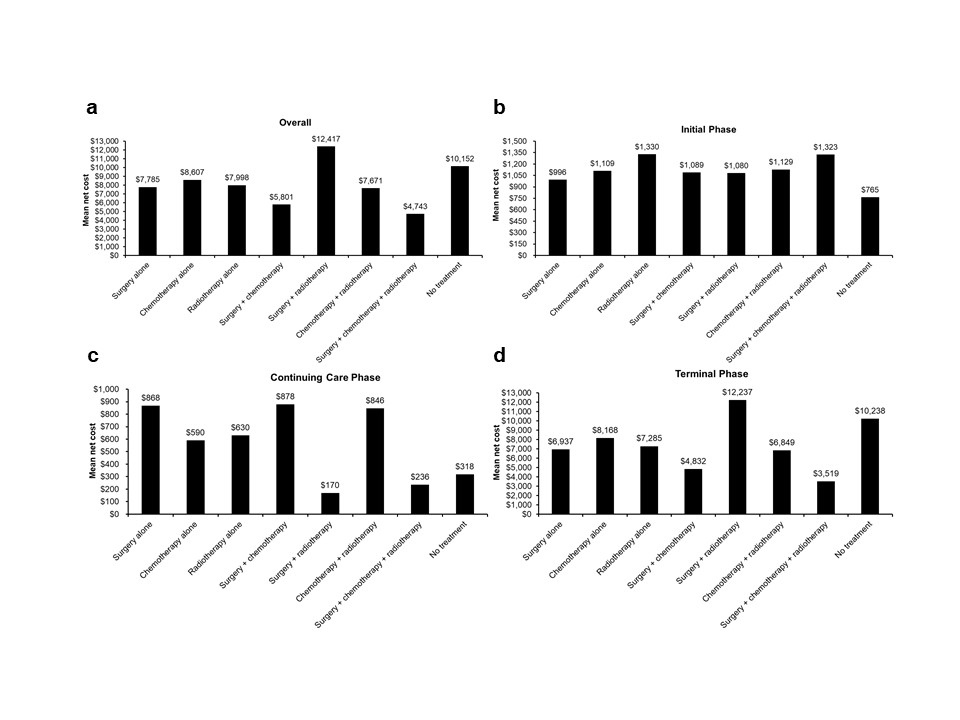

Supplement: Supplementary file 7 — Figure S4a-d. Overall and phase of care net cost of health care resources by treatment type for esophageal adenocarcinoma, 2003–2011: (a) Overall; (b) Initial Phase; (c) Continuing Care Phase; and (d) Terminal Phase. (JPG 80 kb) [file 12885_2018_4620_MOESM7_ESM.jpg]
