# Supplementary material for: Estimates and predictors of health care costs of esophageal adenocarcinoma: a population-based cohort study
Source: BMC Cancer. 2018 Jun 27;18:694. doi: 10.1186/s12885-018-4620-2 (PMC6020438; doi:10.1186/s12885-018-4620-2)
Supplement: Supplementary file 3 — Table S3. Demographic characteristics of esophageal adenocarcinoma cases and controls, 2003–2011. (DOCX 21 kb) [file 12885_2018_4620_MOESM3_ESM.docx]

**Table S3.** Demographic characteristics of esophageal adenocarcinoma cases and controls, 2003-2011

| Variable | Cases N (%) | Controls N (%) |
| --- | --- | --- |
| Total | 3,035 | 560,997 |
| Age group at index date (years) |  |  |
| <50 | 243 (8.0) | 397,807 (70.9) |
| 50-54 | 277 (9.1) | 40,727 (7.3) |
| 55-59 | 333 (11.0) | 33,586 (6.0) |
| 60-64 | 460 (15.2) | 25,986 (4.6) |
| 65-69 | 427 (14.1) | 18,622 (3.3) |
| 70-74 | 416 (13.7) | 15,029 (2.7) |
| 75-79 | 383 (12.6) | 12,153 (2.2) |
| 80-84 | 287 (9.5) | 8,932 (1.6) |
| >85 | 209 (6.9) | 8,155 (1.5) |
| Gender |  |  |
| Female | 499 (16.4) | 283,986 (50.6) |
| Male | 2,536 (83.6) | 277,011 (49.4) |
| Residence |  |  |
| Urban | 2,472 (81.5) | 496,427 (88.5) |
| Rural | 563 (18.6) | 64,564 (11.5) |
| Missing | 0 | 6 (0) |
| Birth country |  |  |
| Outside of Canada | 580 (19.1) |  |
| Canada | 1,860 (61.3) |  |
| Missing | 595 (19.6) |  |
| Income quintile |  |  |
| Q1 (lowest) | 598 (19.7) | 112,242 (20.0) |
| Q2 | 632 (20.8) | 111,694 (19.9) |
| Q3 | 594 (19.6) | 110,634 (19.7) |
| Q4 | 624 (20.6) | 113,287 (20.2) |
| Q5 (highest) | 575 (19.0) | 111,269 (19.8) |
| Missing | 12 (0.4) | 1,871 (0.3) |
| Ontario health region |  |  |
| Erie St. Clair | 153 (5.0) | 27,915 (5.0) |
| South West | 274 (9.0) | 39,655 (7.1) |
| Waterloo Wellington | 174 (5.7) | 30,638 (5.5) |
| Hamilton Niagara Haldimand Brant | 447 (14.7) | 58,963 (10.5) |
| Central West | 101 (3.3) | 35,970 (6.4) |
| Mississauga | 140 (4.6) | 48,689 (8.7) |
| Toronto Central | 202 (6.7) | 52,028 (9.3) |
| Central | 215 (7.1) | 73,176 (13.0) |
| Central East | 342 (11.3) | 66,313 (11.8) |
| South East | 218 (7.2) | 20,678 (3.7) |
| Champlain | 340 (11.2) | 53,156 (9.5) |
| North Simcoe Muskoka | 150 (4.9) | 18,395 (3.3) |
| North East | 196 (6.5) | 24,676 (4.4) |
| North West | 83 (2.7) | 10,745 (1.9) |
| ADGs |  |  |
| 0 | 16 (0.5) | 37,348 (6.7) |
| 1-3 | 119 (3.9) | 75,609 (13.5) |
| 4-7 | 550 (18.1) | 181,988 (32.4) |
| 8-10 | 808 (26.6) | 133,966 (23.9) |
| 11+ | 1,542 (50.8) | 132,086 (23.5) |
| Stage at EAC diagnosis |  |  |
| Stage 0-I | 126 (4.2) |  |
| Stage II | 420 (13.8) |  |
| Stage III | 455 (15.0) |  |
| Stage IV | 940 (31.0) |  |
| Unknown/Missing | 1,094 (36.1) |  |
| EAC treatment |  |  |
| Surgery alone | 537 (17.7) |  |
| Chemotherapy alone | 338 (11.1) |  |
| Radiotherapy alone | 405 (13.3) |  |
| Surgery + chemotherapy | 118 (3.9) |  |
| Surgery + radiotherapy | 6 (0.2) |  |
| Chemotherapy + radiotherapy | 110 (3.6) |  |
| Surgery + chemotherapy + radiotherapy | 7 (0.2) |  |
| No treatment | 1,514 (49.9) |  |
| Index year |  |  |
| 2003 | 285 (9.4) | 59,412 (10.6) |
| 2004 | 291 (9.6) | 60,411 (10.8) |
| 2005 | 283 (9.3) | 61,549 (11.0) |
| 2006 | 324 (10.7) | 61,266 (10.9) |
| 2007 | 300 (9.9) | 61,612 (11.0) |
| 2008 | 351 (11.6) | 62,310 (11.1) |
| 2009 | 387 (12.8) | 63,682 (11.4) |
| 2010 | 401 (13.2) | 65,051 (11.6) |
| 2011 | 413 (13.6) | 65,704 (11.7) |
| Death year |  |  |
| 2003 | 262 (10.5) | 3,093 (16.7) |
| 2004 | 254 (10.2) | 2,748 (14.8) |
| 2005 | 254 (10.2) | 2,591 (14.0) |
| 2006 | 283 (11.4) | 2,305 (12.4) |
| 2007 | 265 (10.6) | 2,032 (11.0) |
| 2008 | 303 (12.2) | 1,832 (9.9) |
| 2009 | 316 (12.7) | 1,564 (8.4) |
| 2010 | 305 (12.3) | 1,309 (7.1) |
| 2011 | 248 (10.0) | 1,062 (5.7) |

ADGs, Aggregated Diagnosis Groups; EAC, esophageal adenocarcinoma.
