# Supplementary material for: Estimates and predictors of health care costs of esophageal adenocarcinoma: a population-based cohort study
Source: BMC Cancer. 2018 Jun 27;18:694. doi: 10.1186/s12885-018-4620-2 (PMC6020438; doi:10.1186/s12885-018-4620-2)
Supplement: Supplementary file 5 — Table S4. Bootstrap samples for mean net costs* of care due to esophageal adenocarcinoma according to service category and phase of care, 2003–2011. (DOCX 13 kb) [file 12885_2018_4620_MOESM5_ESM.docx]

**Table S4** Bootstrap samples for mean net costs^*^ of care due to esophageal adenocarcinoma according to service category and phase of care, 2003-2011

| Service category | Overall | Initial Phase | Continuing Care Phase | Terminal Phase |
| --- | --- | --- | --- | --- |
|  | Mean (95% CI) | Mean (95% CI) | Mean (95% CI) | Mean (95% CI) |
| N | 6,022,000 | 4,227,000 | 3,521,000 | 6,022,000 |
| Outpatient visits | $1,277 ($1,213–$1,341) | $155 ($148–$162) | $117 ($109–$125) | $1,224 ($1,159–$1,290) |
| Emergency department visits | $210 ($188–$232) | $17 ($16–$18) | $9 ($8–$11) | $208 ($186–$230) |
| Same-day surgery | $179 ($164–$193) | $29 ($27–$31) | $27 ($24–$30) | $168 ($153–$182) |
| Inpatient hospitalization | $5,493 ($5,128–$5,858) | $455 ($416–$494) | $342 ($296–$389) | $5,289 ($4,922–$5,655) |
| Medications | $207 ($190–$225) | $46 ($41–$51) | $22 ($14–$30) | $224 ($207–$240) |
| Home care | $479 ($450 –$508) | $79 ($72–$86) | $47 ($39–$55) | $449 ($420–$477) |
| Continuing care | -$32 (-$47– -$16) | -$7 (-$11– -$2) | -$18 (-$25– -$12) | -$3 (-$15–$10) |
| Long-term care | -$43 (-$63– -$23) | -$11 (-$17– -$4) | -$24 (-$33– -$15) | $1 (-$15–$16) |
| Total net costs | $8,992 ($8,538–$9,445) | $1,017 ($955–$1,079) | $667 ($593–$741) | $8,667 ($8,207–$9,127) |

Number of resamples: 1000. Net costs of care due to esophageal adenocarcinoma were generated using generalized estimating equations. ^*^Mean health care costs are expressed in 2016 Canadian dollars per 30 patient-days. EAC, esophageal adenocarcinoma; CI, confidence intervals.
