# Supplementary material for: Estimates and predictors of health care costs of esophageal adenocarcinoma: a population-based cohort study
Source: BMC Cancer. 2018 Jun 27;18:694. doi: 10.1186/s12885-018-4620-2 (PMC6020438; doi:10.1186/s12885-018-4620-2)
Supplement: Supplementary file 8 — Table S5. Mean net costs* of care due to esophageal adenocarcinoma according to service category and phase of care, 2003–2011: Sensitivity analysis. Table S6. Overall and phase of care net cost of health care resources by stage at diagnosis and treatment type for the esophageal adenocarcinoma cohort, 2003–2011: Sensitivity analysis. Table S7. Predictors of total net costs of care in individuals with a diagnosis of esophageal adenocarcinoma according to phase of care, 2003–2011: Multivariate generalized estimation equations with a log link function and gamma distribution – Sensitivity analysis. (DOCX 37 kb) [file 12885_2018_4620_MOESM8_ESM.docx]

**Table S5** Mean net costs^*^ of care due to esophageal adenocarcinoma according to service category and phase of care, 2003-2011: Sensitivity analysis

| Service category | Overall | Initial Phase^†^ | Continuing Care Phase | Terminal Phase^‡^ |
| --- | --- | --- | --- | --- |
|  | Mean (95% CI) | Mean (95% CI) | Mean (95% CI) | Mean (95% CI) |
| N | 6,022 | 4,880 | 4,227 | 6,022 |
| Outpatient visits | $1,279 ($1,215–$1,343) | $208 ($199–$217) | $195 ($186–$204) | $1,098 ($1,032–$1,165) |
| Emergency department visits | $209 ($188–$231) | $26 ($25–$28) | $18 ($16–$20) | $192 ($170–$214) |
| Same-day surgery | $179 ($164–$193) | $35 ($33–$37) | $39 ($37–$42) | $146 ($131–$160) |
| Inpatient hospitalization | $5,501 ($5,135–$5,867) | $660 ($610–$710) | $600 ($544–$657) | $4,893 ($4,524–$5,261) |
| Medications | $207 ($190–$225) | $68 ($63–$74) | $37 ($29–$45) | $184 ($169–$199) |
| Home care | $479 ($450 –$509) | $122 ($113–$130) | $97 ($87–$106) | $374 ($347–$401) |
| Continuing care | -$32 (-$47– -$17) | -$2 (-$6–$2) | -$26 (-$35– -$18) | $1 (-$10–$12) |
| Long-term care | -$44 (-$64– -$24) | -$4 (-$9–$1) | -$38 (-$49– -$26) | $8 (-$6–$21) |
| Total net costs | $9,002 ($8,547–$9,456) | $1,447 ($1,373–$1,520) | $1,236 ($1,148–$1,323) | $7,797 ($7,331–$8,263) |

Net costs of care due to esophageal adenocarcinoma were generated using generalized estimating equations. ^*^Mean health care costs are expressed in 2016 Canadian dollars per 30 patient-days. ^†^Initial Phase: 6 months after diagnosis. ^‡^Terminal Phase: 6 months before death. EAC, esophageal adenocarcinoma; CI, confidence intervals.

**Table S6** Overall and phase of care net cost of health care resources by stage at diagnosis and treatment type for the esophageal adenocarcinoma cohort, 2003-2011: Sensitivity analysis

| Cost category | Overall |  | Initial Phase^†^ |  | Continuing Care Phase |  | ^‡^Terminal Phase |
| --- | --- | --- | --- | --- | --- | --- | --- |
|  | Mean^*^ (95% CI) | N (Cases) | Mean^*^ (95% CI) | N (Cases) | Mean^*^ (95% CI) | N (Cases) | Mean^*^ (95% CI) |
| Stage at EAC diagnosis |  |  |  |  |  |  |  |
| Stage 0-I | $5,094 ($2,659‒$7,529) | 116 | $1,014 ($779‒$1,250) | 102 | $1,120 ($904‒$1,337) | 126 | $3,325 ($887‒$5,763) |
| Stage II | $6,192 ($5,368‒$7,015) | 361 | $1,361 ($1,180‒$1,542) | 282 | $1,293 ($1,162‒$1,424) | 416 | $4,260 ($3,428‒$5,092) |
| Stage III | $7,413 ($6,679‒$8,147) | 369 | $1,874 ($1,687‒$2,061) | 273 | $1,485 ($1,350‒$1,621) | 451 | $5,139 ($4,381‒$5,897) |
| Stage IV | $9,978 ($9,093‒$10,864) | 452 | $1,529 ($1,407‒$1,651) | 222 | $1,054 ($906‒$1,202) | 930 | $9,306 ($8,398‒$10,214) |
| Type of EAC treatment |  |  |  |  |  |  |  |
| Surgery alone | $7,785 ($6,667‒$8,903) | 452 | $1,379 ($1,218‒$1,539) | 361 | $1,416 ($1,190‒$1,641) | 533 | $5,785 ($4,650‒$6,919) |
| Chemotherapy alone | $8,607 ($7,565‒$9,650) | 216 | $1,465 ($1,243‒$1,686) | 161 | $1,312 ($1,134‒$1,490) | 338 | $7,268 ($6,173‒$8,363) |
| Radiotherapy alone | $7,998 ($7,095‒$8,901) | 304 | $1,660 ($1,444‒$1,876) | 228 | $1,540 ($1,387‒$1,693) | 405 | $6,065 ($5,126‒$7,004) |
| Surgery + chemotherapy | $5,801 ($4,751‒$6,850) | 105 | $1,338 ($1,040‒$1,636) | 81 | $1,568 ($1,280‒$1,856) | 118 | $3,654 ($2,578‒$4,731) |
| Surgery + radiotherapy | $12,417 ($2,067‒$22,767) | ‒ | $2,246 ($527‒$3,965) | ‒ | $1,256 ($300‒$2,212) | 6 | $10,770 (-$348‒$21,889) |
| Chemotherapy + radiotherapy | $7,671 ($6,023‒$9320) | 90 | $1,633 ($1,277‒$1,989) | 68 | $1,528 ($1,268‒$1,788) | 109 | $5,520 ($3,815‒$7,225) |
| Surgery + chemotherapy + radiotherapy | $4,743 ($1,755‒$7,731) | 7 | $1,834 ($413‒$3,255) | 7 | $1,215 ($762‒$1,669) | 7 | $1,694 ($272‒$3,117) |
| No treatment | $10,152 ($9,431‒$10,873) | 730 | $1,380 ($1,283‒1,476) | 377 | $723 ($632‒$815) | 1,495 | $9,613 ($8,876‒$10,350) |

^*^Mean health care costs are expressed in 2016 Canadian dollars per 30 patient-days. ^†^Initial Phase: 6 months after diagnosis. ^‡^Terminal Phase: 6 months before death. ‘–’, counts less than six are suppressed. EAC, esophageal adenocarcinoma; CI, confidence intervals

**Table S7** Predictors of total net costs of care in individuals with a diagnosis of esophageal adenocarcinoma according to phase of care, 2003-2011: Multivariate generalized estimation equations with a log link function and gamma distribution – Sensitivity analysis

| Variable | Initial Phase | | | Continuing Care Phase | | | Terminal Phase | | |  |
| --- | --- | --- | --- | --- | --- | --- | --- | --- | --- | --- |
|  | Estimate | 95% CI | *P*-value | Estimate | 95% CI | *P*-value | Estimate | 95% CI | *P*-value | |
| Intercept | 6.329 | 5.962–6.729 | <0.001 | 5.638 | 4.955–6.426 | <0.001 | 7.417 | 6.796–8.071 | <0.001 | |
| Age group at index date (years)^*^ |  |  |  |  |  |  |  |  |  | |
| <50 |  |  |  | Reference |  |  | Reference |  |  | |
| 50-54 |  |  |  | -0.044 | -0.233–0.144 | 0.646 | -0.009 | -0.257–0.237 | 0.941 | |
| 55-59 |  |  |  | -0.099 | -0.284–0.085 | 0.294 | -0.216 | -0.456–0.022 | 0.077 | |
| 60-64 |  |  |  | -0.073 | -0.249–0.100 | 0.412 | 0.023 | -0.207–0.249 | 0.842 | |
| 65-69 |  |  |  | 0.057 | -0.123–0.234 | 0.533 | -0.106 | -0.339–0.124 | 0.370 | |
| 70-74 |  |  |  | 0.067 | -0.124–0.256 | 0.491 | 0.355 | 0.115–0.592 | 0.004 | |
| 75-79 |  |  |  | -0.186 | -0.380–0.007 | 0.059 | 0.174 | -0.069–0.414 | 0.158 | |
| 80-84 |  |  |  | -0.183 | -0.411–0.049 | 0.119 | 0.113 | -0.151–0.377 | 0.403 | |
| >85 |  |  |  | -0.408 | -0.690– -0.115 | 0.005 | 0.282 | -0.059–0.634 | 0.110 | |
| Ontario health region^**^ |  |  |  |  |  |  |  |  |  | |
| Central |  |  |  |  |  |  | Reference |  |  | |
| Erie St. Clair |  |  |  |  |  |  | 0.131 | -0.197–0.463 | 0.437 | |
| South West |  |  |  |  |  |  | 0.155 | -0.133–0.440 | 0.289 | |
| Waterloo Wellington |  |  |  |  |  |  | 0.356 | 0.048–0.664 | 0.023 | |
| Hamilton Niagara Haldimand Brant |  |  |  |  |  |  | 0.065 | -0.194–0.316 | 0.618 | |
| Central West |  |  |  |  |  |  | -0.237 | -0.634–0.182 | 0.254 | |
| Mississauga |  |  |  |  |  |  | 0.221 | -0.163–0.622 | 0.269 | |
| Toronto Central |  |  |  |  |  |  | 0.162 | -0.149–0.475 | 0.308 | |
| Central East |  |  |  |  |  |  | -0.141 | -0.421–0.134 | 0.319 | |
| South East |  |  |  |  |  |  | -0.104 | -0.395–0.184 | 0.480 | |
| Champlain |  |  |  |  |  |  | 0.075 | -0.196–0.341 | 0.583 | |
| North Simcoe Muskoka |  |  |  |  |  |  | 0.009 | -0.316–0.338 | 0.956 | |
| North East |  |  |  |  |  |  | 0.446 | 0.128–0.765 | 0.006 | |
| North West |  |  |  |  |  |  | 0.391 | 0.024–0.770 | 0.040 | |
| ADGs^‡^ |  |  |  |  |  |  |  |  |  | |
| 0 |  |  |  | Reference |  |  |  |  |  | |
| 1-3 |  |  |  | 0.694 | -0.047–1.322 | 0.044 |  |  |  | |
| 4-7 |  |  |  | 0.781 | 0.061–1.376 | 0.019 |  |  |  | |
| 8-10 |  |  |  | 0.832 | 0.113–1.427 | 0.012 |  |  |  | |
| 11+ |  |  |  | 0.969 | 0.253–1.561 | 0.003 |  |  |  | |
| Stage at EAC diagnosis^§^ |  |  |  |  |  |  |  |  |  | |
| Stage 0-I | Reference |  |  | Reference |  |  | Reference |  |  | |
| Stage II | 0.286 | 0.087–0.479 | 0.004 | 0.137 | -0.013–0.284 | 0.070 | 0.290 | -1.113–2.48 | 0.735 | |
| Stage III | 0.627 | 0.429–0.819 | <0.001 | 0.231 | 0.080–0.378 | 0.002 | 1.052 | 0.542–1.532 | <.0001 | |
| Stage IV | 0.421 | 0.226–0.609 | <0.001 | 0.114 | -0.048–0.274 | 0.1631 | 2.004 | 0.815–3.700 | 0.004 | |
| EAC treatment^¶^ |  |  |  |  |  |  |  |  |  | |
| No treatment |  |  |  | Reference |  |  | Reference |  |  | |
| Surgery alone |  |  |  | 0.412 | 0.284–0.539 | <0.001 | -4.649 | -7.471– -0.901 | 0.001 | |
| Chemotherapy alone |  |  |  | 0.368 | 0.216–0.523 | <0.001 | -1.532 | -3.655–0.605 | 0.139 | |
| Radiotherapy alone |  |  |  | 0.466 | 0.331–0.601 | <0.001 | -0.960 | -3.151–1.513 | 0.386 | |
| Surgery + chemotherapy |  |  |  | 0.564 | 0.361–0.775 | <0.001 | -1.643 | -2.473– -0.688 | 0.000 | |
| Surgery + radiotherapy |  |  |  | 0.848 | -0.218–2.442 | 0.192 | -0.563 | -2.249–2.925 | 0.626 | |
| Chemotherapy + radiotherapy |  |  |  | 0.629 | 0.424–0.841 | <0.001 | -1.852 | -4.469–1.910 | 0.202 | |
| Surgery + chemotherapy + radiotherapy |  |  |  | 0.551 | -0.046–1.271 | 0.097 | -1.220 | -2.795–1.258 | 0.213 | |
| Year of EAC diagnosis^€^ |  |  |  |  |  |  |  |  |  | |
| 2003 | Reference |  |  | Reference |  |  | Reference |  |  | |
| 2004 | 0.155 | -0.247–0.528 | 0.431 | 0.207 | -0.129–0.524 | 0.214 | -0.109 | -0.539–0.293 | 0.606 | |
| 2005 | 0.476 | 0.075–0.849 | 0.016 | 0.357 | 0.018–0.679 | 0.034 | 0.482 | 0.054–0.881 | 0.022 | |
| 2006 | 0.590 | 0.201–0.948 | 0.002 | 0.530 | 0.203–0.838 | 0.001 | 0.334 | -0.083–0.718 | 0.102 | |
| 2007 | 0.639 | 0.247–1.000 | 0.001 | 0.312 | -0.016–0.621 | 0.054 | 0.457 | 0.037–0.846 | 0.026 | |
| 2008 | 0.654 | 0.266–1.009 | 0.001 | 0.583 | 0.260–0.885 | <0.001 | 0.567 | 0.154–0.947 | 0.005 | |
| 2009 | 0.811 | 0.427–1.162 | <0.001 | 0.563 | 0.240–0.864 | <0.001 | 0.672 | 0.261–1.051 | 0.001 | |
| 2010 | 0.721 | 0.335–1.073 | <0.001 | 0.415 | 0.092–0.716 | 0.009 | 0.527 | 0.116–0.906 | 0.009 | |
| 2011 | 0.819 | 0.431–1.173 | <0.001 | 0.461 | 0.136–0.766 | 0.004 | 0.541 | 0.123–0.928 | 0.008 | |
| Income quintile-Comorbidity interaction^††^ |  |  |  |  |  |  |  |  |  | |
| Q1 (lowest)*ADGs |  |  |  |  |  |  | -0.026 | -0.193–0.142 | 0.757 | |
| Q2*ADGs |  |  |  |  |  |  | -0.120 | -0.198–-0.041 | 0.003 | |
| Q3*ADGs |  |  |  |  |  |  | 0.010 | -0.057–0.078 | 0.765 | |
| Q4*ADGs |  |  |  |  |  |  | -0.067 | -0.122– -0.013 | 0.016 | |
| Comorbidity-EAC stage interaction^‡‡^ |  |  |  |  |  |  |  |  |  | |
| ADGs 1-3*EAC stage II |  |  |  |  |  |  | 1.099 | -1.196–2.745 | 0.246 | |
| ADGs 1-3*EAC stage III |  |  |  |  |  |  | -0.468 | -1.298–0.395 | 0.276 | |
| ADGs 1-3*EAC stage IV |  |  |  |  |  |  | -0.791 | -2.529–0.487 | 0.283 | |
| ADGs 4-7*EAC stage II |  |  |  |  |  |  | 0.277 | -1.910–1.669 | 0.746 | |
| ADGs 4-7*EAC stage III |  |  |  |  |  |  | -0.075 | -0.523–0.376 | 0.742 | |
| ADGs 4-7*EAC stage IV |  |  |  |  |  |  | -0.481 | -2.152–0.649 | 0.481 | |
| ADGs 8-10*EAC stage II |  |  |  |  |  |  | 0.185 | -1.993–1.556 | 0.827 | |
| ADGs 8-10*EAC stage III |  |  |  |  |  |  | -0.308 | -0.702–0.086 | 0.125 | |
| ADGs 8-10*EAC stage IV |  |  |  |  |  |  | -0.888 | -2.552–0.228 | 0.190 | |
| ADGs 11+*EAC stage II |  |  |  |  |  |  | 0.246 | -1.924–1.596 | 0.769 | |
| ADGs 11+*EAC stage IV |  |  |  |  |  |  | -0.607 | -2.268–0.501 | 0.368 | |
| Comorbidity-EAC treatment interaction^‡‡‡^ |  |  |  |  |  |  |  |  |  | |
| ADGs 1-3*Surgery alone |  |  |  |  |  |  | 4.613 | 0.848–7.464 | 0.002 | |
| ADGs 1-3*Chemotherapy alone |  |  |  |  |  |  | 0.515 | -1.719–2.810 | 0.643 | |
| ADGs 1-3*Radiotherapy alone |  |  |  |  |  |  | 1.198 | -1.303–3.434 | 0.289 | |
| ADGs 1-3*Surgery + chemotherapy |  |  |  |  |  |  | -1.725 | -3.675–1.833 | 0.163 | |
| ADGs 1-3*Chemotherapy + radiotherapy |  |  |  |  |  |  | 2.380 | -1.361–5.018 | 0.099 | |
| ADGs 4-7*Surgery alone |  |  |  |  |  |  | 4.017 | 0.279–6.820 | 0.006 | |
| ADGs 4-7*Chemotherapy alone |  |  |  |  |  |  | 1.269 | -0.749–3.288 | 0.191 | |
| ADGs 4-7*Radiotherapy alone |  |  |  |  |  |  | 1.199 | -1.219–3.321 | 0.262 | |
| ADGs 4-7*Surgery + chemotherapy |  |  |  |  |  |  | -0.133 | -0.908–0.707 | 0.745 | |
| ADGs 4-7*Surgery + radiotherapy |  |  |  |  |  |  | -1.180 | -5.069–2.710 | 0.474 | |
| ADGs 4-7*Chemotherapy + radiotherapy |  |  |  |  |  |  | 2.448 | -1.228–4.917 | 0.075 | |
| ADGs 4-7*Surgery + chemotherapy + radiotherapy |  |  |  |  |  |  | 0.316 | -2.211–2.845 | 0.788 | |
| ADGs 8-10*Surgery alone |  |  |  |  |  |  | 4.305 | 0.572–7.100 | 0.003 | |
| ADGs 8-10*Chemotherapy alone |  |  |  |  |  |  | 1.452 | -0.554–3.458 | 0.131 | |
| ADGs 8-10*Radiotherapy alone |  |  |  |  |  |  | 0.815 | -1.598–2.931 | 0.445 | |
| AADGs 8-10*Surgery + chemotherapy |  |  |  |  |  |  | 0.280 | -0.421–1.011 | 0.442 | |
| ADGs 8-10*Surgery + radiotherapy |  |  |  |  |  |  | 0.741 | -3.123–4.606 | 0.649 | |
| ADGs 8-10*Chemotherapy + radiotherapy |  |  |  |  |  |  | 3.391 | -0.276–5.836 | 0.013 | |
| ADGs 11+*Surgery alone |  |  |  |  |  |  | 4.730 | 1.003–7.515 | 0.001 | |
| ADGs 11+*Chemotherapy alone |  |  |  |  |  |  | 1.253 | -0.738–3.244 | 0.189 | |
| ADGs 11+*Radiotherapy alone |  |  |  |  |  |  | 1.085 | -1.324–3.196 | 0.307 | |
| ADGs 11+*Chemotherapy + radiotherapy |  |  |  |  |  |  | 2.689 | -0.972–5.122 | 0.048 | |
| EAC stage-EAC treatment interaction^§§^ |  |  |  |  |  |  |  |  |  | |
| EAC stage II*Surgery alone |  |  |  |  |  |  | 0.054 | -0.587–0.706 | 0.869 | |
| EAC stage II*Chemotherapy alone |  |  |  |  |  |  | 0.126 | -0.853–1.020 | 0.790 | |
| EAC stage II*Radiotherapy alone |  |  |  |  |  |  | -0.292 | -1.047–0.443 | 0.440 | |
| EAC stage II*Surgery + chemotherapy |  |  |  |  |  |  | 0.832 | -0.270–1.864 | 0.122 | |
| EAC stage II*Chemotherapy + radiotherapy |  |  |  |  |  |  | -1.371 | -2.629– -0.278 | 0.020 | |
| EAC stage III*Surgery alone |  |  |  |  |  |  | -0.308 | -0.938–0.329 | 0.339 | |
| EAC stage III*Chemotherapy alone |  |  |  |  |  |  | -0.198 | -1.174–0.692 | 0.675 | |
| EAC stage III*Radiotherapy alone |  |  |  |  |  |  | -0.359 | -1.106–0.367 | 0.337 | |
| EAC stage III*Surgery + chemotherapy |  |  |  |  |  |  | 1.375 | 0.272–2.404 | 0.011 | |
| EAC stage III*Chemotherapy + radiotherapy |  |  |  |  |  |  | -0.828 | -2.099–0.280 | 0.165 | |
| EAC stage III*Surgery + chemotherapy + radiotherapy |  |  |  |  |  |  | 0.359 | -2.198–2.920 | 0.764 | |
| EAC stage IV*Surgery alone |  |  |  |  |  |  | -0.153 | -0.821–0.531 | 0.656 | |
| EAC stage IV*Chemotherapy alone |  |  |  |  |  |  | 0.075 | -0.854–0.903 | 0.865 | |
| EAC stage IV*Radiotherapy alone |  |  |  |  |  |  | -0.087 | -0.807–0.611 | 0.808 | |
| EAC stage IV*Surgery + chemotherapy |  |  |  |  |  |  | 1.704 | 0.565–2.800 | 0.003 | |
| EAC stage IV*Chemotherapy + radiotherapy |  |  |  |  |  |  | -1.222 | -2.467– -0.148 | 0.035 | |

Initial Phase: final multivariable analysis adjusted for stage at EAC diagnosis and year of EAC diagnosis (index date). Overall *P* values (adjusted): ^§^stage at EAC diagnosis: *P* < 0.001; ^€^year of EAC diagnosis: *P* < 0.001.

Continuing Care Phase: final multivariable analysis adjusted for age group at index date, comorbidity, measured by the Johns Hopkins ADGs, stage at EAC diagnosis, treatment for EAC, and year of EAC diagnosis (index date). Overall *P* values (adjusted): ^*^age group at index date: *P* = 0.007; ^‡^comorbidity: *P* < 0.001; ^§^stage at EAC diagnosis: *P* = 0.019; ^¶^treatment for EAC: *P* < 0.001; ^€^year of EAC diagnosis: *P* < 0.001.

Terminal Phase: final multivariable analysis adjusted for age group at index date, Ontario health region, stage at EAC diagnosis, treatment for EAC, year of EAC diagnosis (index date), income quintile and comorbidity interaction, comorbidity and stage at EAC diagnosis interaction, comorbidity and EAC treatment interaction, and stage at EAC diagnosis and EAC treatment interaction. Overall *P* values (adjusted): ^*^age group at index date: *P* < 0.001; ^**^Ontario health region: *P* < 0.001; ^§^stage at EAC diagnosis: *P* < 0.001; ^¶^treatment for EAC: *P* = 0.012; ^€^year of EAC diagnosis: *P* < 0.001; ^††^Income quintile-Comorbidity interaction: *P* = 0.007; ^‡‡^Comorbidity-EAC stage interaction: *P* = 0.011; ^‡‡‡^Comorbidity- EAC treatment interaction: *P* < 0.001; ^§§^EAC stage-EAC treatment interaction: *P* < 0.001.

CI, confidence interval; ADGs, Aggregated Diagnosis Groups; EAC, esophageal adenocarcinoma.
